# The landscape of viral associations in human cancers

**DOI:** 10.1038/s41588-019-0558-9

**Published:** 2020-02-05

**Authors:** Marc Zapatka, Ivan Borozan, Daniel S. Brewer, Murat Iskar, Adam Grundhoff, Malik Alawi, Nikita Desai, Holger Sültmann, Holger Moch, PCAWG Pathogens, Malik Alawi, Ivan Borozan, Daniel S. Brewer, Colin S. Cooper, Nikita Desai, Roland Eils, Vincent Ferretti, Adam Grundhoff, Murat Iskar, Kortine Kleinheinz, Peter Lichter, Hidewaki Nakagawa, Akinyemi I. Ojesina, Chandra Sekhar Pedamallu, Matthias Schlesner, Xiaoping Su, Marc Zapatka, Colin S. Cooper, Roland Eils, Vincent Ferretti, Peter Lichter, PCAWG Consortium, Lauri A. Aaltonen, Federico Abascal, Adam Abeshouse, Hiroyuki Aburatani, David J. Adams, Nishant Agrawal, Keun Soo Ahn, Sung-Min Ahn, Hiroshi Aikata, Rehan Akbani, Kadir C. Akdemir, Hikmat Al-Ahmadie, Sultan T. Al-Sedairy, Fatima Al-Shahrour, Malik Alawi, Monique Albert, Kenneth Aldape, Ludmil B. Alexandrov, Adrian Ally, Kathryn Alsop, Eva G. Alvarez, Fernanda Amary, Samirkumar B. Amin, Brice Aminou, Ole Ammerpohl, Matthew J. Anderson, Yeng Ang, Davide Antonello, Pavana Anur, Samuel Aparicio, Elizabeth L. Appelbaum, Yasuhito Arai, Axel Aretz, Koji Arihiro, Shun-ichi Ariizumi, Joshua Armenia, Laurent Arnould, Sylvia Asa, Yassen Assenov, Gurnit Atwal, Sietse Aukema, J. Todd Auman, Miriam R. R. Aure, Philip Awadalla, Marta Aymerich, Gary D. Bader, Adrian Baez-Ortega, Matthew H. Bailey, Peter J. Bailey, Miruna Balasundaram, Saianand Balu, Pratiti Bandopadhayay, Rosamonde E. Banks, Stefano Barbi, Andrew P. Barbour, Jonathan Barenboim, Jill Barnholtz-Sloan, Hugh Barr, Elisabet Barrera, John Bartlett, Javier Bartolome, Claudio Bassi, Oliver F. Bathe, Daniel Baumhoer, Prashant Bavi, Stephen B. Baylin, Wojciech Bazant, Duncan Beardsmore, Timothy A. Beck, Sam Behjati, Andreas Behren, Beifang Niu, Cindy Bell, Sergi Beltran, Christopher Benz, Andrew Berchuck, Anke K. Bergmann, Erik N. Bergstrom, Benjamin P. Berman, Daniel M. Berney, Stephan H. Bernhart, Rameen Beroukhim, Mario Berrios, Samantha Bersani, Johanna Bertl, Miguel Betancourt, Vinayak Bhandari, Shriram G. Bhosle, Andrew V. Biankin, Matthias Bieg, Darell Bigner, Hans Binder, Ewan Birney, Michael Birrer, Nidhan K. Biswas, Bodil Bjerkehagen, Tom Bodenheimer, Lori Boice, Giada Bonizzato, Johann S. De Bono, Arnoud Boot, Moiz S. Bootwalla, Ake Borg, Arndt Borkhardt, Keith A. Boroevich, Ivan Borozan, Christoph Borst, Marcus Bosenberg, Mattia Bosio, Jacqueline Boultwood, Guillaume Bourque, Paul C. Boutros, G. Steven Bova, David T. Bowen, Reanne Bowlby, David D. L. Bowtell, Sandrine Boyault, Rich Boyce, Jeffrey Boyd, Alvis Brazma, Paul Brennan, Daniel S. Brewer, Arie B. Brinkman, Robert G. Bristow, Russell R. Broaddus, Jane E. Brock, Malcolm Brock, Annegien Broeks, Angela N. Brooks, Denise Brooks, Benedikt Brors, Søren Brunak, Timothy J. C. Bruxner, Alicia L. Bruzos, Alex Buchanan, Ivo Buchhalter, Christiane Buchholz, Susan Bullman, Hazel Burke, Birgit Burkhardt, Kathleen H. Burns, John Busanovich, Carlos D. Bustamante, Adam P. Butler, Atul J. Butte, Niall J. Byrne, Anne-Lise Børresen-Dale, Samantha J. Caesar-Johnson, Andy Cafferkey, Declan Cahill, Claudia Calabrese, Carlos Caldas, Fabien Calvo, Niedzica Camacho, Peter J. Campbell, Elias Campo, Cinzia Cantù, Shaolong Cao, Thomas E. Carey, Joana Carlevaro-Fita, Rebecca Carlsen, Ivana Cataldo, Mario Cazzola, Jonathan Cebon, Robert Cerfolio, Dianne E. Chadwick, Dimple Chakravarty, Don Chalmers, Calvin Wing Yiu Chan, Kin Chan, Michelle Chan-Seng-Yue, Vishal S. Chandan, David K. Chang, Stephen J. Chanock, Lorraine A. Chantrill, Aurélien Chateigner, Nilanjan Chatterjee, Kazuaki Chayama, Hsiao-Wei Chen, Jieming Chen, Ken Chen, Yiwen Chen, Zhaohong Chen, Andrew D. Cherniack, Jeremy Chien, Yoke-Eng Chiew, Suet-Feung Chin, Juok Cho, Sunghoon Cho, Jung Kyoon Choi, Wan Choi, Christine Chomienne, Zechen Chong, Su Pin Choo, Angela Chou, Angelika N. Christ, Elizabeth L. Christie, Eric Chuah, Carrie Cibulskis, Kristian Cibulskis, Sara Cingarlini, Peter Clapham, Alexander Claviez, Sean Cleary, Nicole Cloonan, Marek Cmero, Colin C. Collins, Ashton A. Connor, Susanna L. Cooke, Colin S. Cooper, Leslie Cope, Vincenzo Corbo, Matthew G. Cordes, Stephen M. Cordner, Isidro Cortés-Ciriano, Kyle Covington, Prue A. Cowin, Brian Craft, David Craft, Chad J. Creighton, Yupeng Cun, Erin Curley, Ioana Cutcutache, Karolina Czajka, Bogdan Czerniak, Rebecca A. Dagg, Ludmila Danilova, Maria Vittoria Davi, Natalie R. Davidson, Helen Davies, Ian J. Davis, Brandi N. Davis-Dusenbery, Kevin J. Dawson, Francisco M. De La Vega, Ricardo De Paoli-Iseppi, Timothy Defreitas, Angelo P. Dei Tos, Olivier Delaneau, John A. Demchok, Jonas Demeulemeester, German M. Demidov, Deniz Demircioğlu, Nening M. Dennis, Robert E. Denroche, Stefan C. Dentro, Nikita Desai, Vikram Deshpande, Amit G. Deshwar, Christine Desmedt, Jordi Deu-Pons, Noreen Dhalla, Neesha C. Dhani, Priyanka Dhingra, Rajiv Dhir, Anthony DiBiase, Klev Diamanti, Li Ding, Shuai Ding, Huy Q. Dinh, Luc Dirix, HarshaVardhan Doddapaneni, Nilgun Donmez, Michelle T. Dow, Ronny Drapkin, Oliver Drechsel, Ruben M. Drews, Serge Serge, Tim Dudderidge, Ana Dueso-Barroso, Andrew J. Dunford, Michael Dunn, Lewis Jonathan Dursi, Fraser R. Duthie, Ken Dutton-Regester, Jenna Eagles, Douglas F. Easton, Stuart Edmonds, Paul A. Edwards, Sandra E. Edwards, Rosalind A. Eeles, Anna Ehinger, Juergen Eils, Roland Eils, Adel El-Naggar, Matthew Eldridge, Kyle Ellrott, Serap Erkek, Georgia Escaramis, Shadrielle M. G. Espiritu, Xavier Estivill, Dariush Etemadmoghadam, Jorunn E. Eyfjord, Bishoy M. Faltas, Daiming Fan, Yu Fan, William C. Faquin, Claudiu Farcas, Matteo Fassan, Aquila Fatima, Francesco Favero, Nodirjon Fayzullaev, Ina Felau, Sian Fereday, Martin L. Ferguson, Vincent Ferretti, Lars Feuerbach, Matthew A. Field, J. Lynn Fink, Gaetano Finocchiaro, Cyril Fisher, Matthew W. Fittall, Anna Fitzgerald, Rebecca C. Fitzgerald, Adrienne M. Flanagan, Neil E. Fleshner, Paul Flicek, John A. Foekens, Kwun M. Fong, Nuno A. Fonseca, Christopher S. Foster, Natalie S. Fox, Michael Fraser, Scott Frazer, Milana Frenkel-Morgenstern, William Friedman, Joan Frigola, Catrina C. Fronick, Akihiro Fujimoto, Masashi Fujita, Masashi Fukayama, Lucinda A. Fulton, Robert S. Fulton, Mayuko Furuta, P. Andrew Futreal, Anja Füllgrabe, Stacey B. Gabriel, Steven Gallinger, Carlo Gambacorti-Passerini, Jianjiong Gao, Shengjie Gao, Levi Garraway, Øystein Garred, Erik Garrison, Dale W. Garsed, Nils Gehlenborg, Josep L. L. Gelpi, Joshy George, Daniela S. Gerhard, Clarissa Gerhauser, Jeffrey E. Gershenwald, Mark Gerstein, Moritz Gerstung, Gad Getz, Mohammed Ghori, Ronald Ghossein, Nasra H. Giama, Richard A. Gibbs, Bob Gibson, Anthony J. Gill, Pelvender Gill, Dilip D. Giri, Dominik Glodzik, Vincent J. Gnanapragasam, Maria Elisabeth Goebler, Mary J. Goldman, Carmen Gomez, Santiago Gonzalez, Abel Gonzalez-Perez, Dmitry A. Gordenin, James Gossage, Kunihito Gotoh, Ramaswamy Govindan, Dorthe Grabau, Janet S. Graham, Robert C. Grant, Anthony R. Green, Eric Green, Liliana Greger, Nicola Grehan, Sonia Grimaldi, Sean M. Grimmond, Robert L. Grossman, Adam Grundhoff, Gunes Gundem, Qianyun Guo, Manaswi Gupta, Shailja Gupta, Ivo G. Gut, Marta Gut, Jonathan Göke, Gavin Ha, Andrea Haake, David Haan, Siegfried Haas, Kerstin Haase, James E. Haber, Nina Habermann, Faraz Hach, Syed Haider, Natsuko Hama, Freddie C. Hamdy, Anne Hamilton, Mark P. Hamilton, Leng Han, George B. Hanna, Martin Hansmann, Nicholas J. Haradhvala, Olivier Harismendy, Ivon Harliwong, Arif O. Harmanci, Eoghan Harrington, Takanori Hasegawa, David Haussler, Steve Hawkins, Shinya Hayami, Shuto Hayashi, D. Neil Hayes, Stephen J. Hayes, Nicholas K. Hayward, Steven Hazell, Yao He, Allison P. Heath, Simon C. Heath, David Hedley, Apurva M. Hegde, David I. Heiman, Michael C. Heinold, Zachary Heins, Lawrence E. Heisler, Eva Hellstrom-Lindberg, Mohamed Helmy, Seong Gu Heo, Austin J. Hepperla, José María Heredia-Genestar, Carl Herrmann, Peter Hersey, Julian M. Hess, Holmfridur Hilmarsdottir, Jonathan Hinton, Satoshi Hirano, Nobuyoshi Hiraoka, Katherine A. Hoadley, Asger Hobolth, Ermin Hodzic, Jessica I. Hoell, Steve Hoffmann, Oliver Hofmann, Andrea Holbrook, Aliaksei Z. Holik, Michael A. Hollingsworth, Oliver Holmes, Robert A. Holt, Chen Hong, Eun Pyo Hong, Jongwhi H. Hong, Gerrit K. Hooijer, Henrik Hornshøj, Fumie Hosoda, Yong Hou, Volker Hovestadt, William Howat, Alan P. Hoyle, Ralph H. Hruban, Jianhong Hu, Taobo Hu, Xing Hua, Kuan-lin Huang, Mei Huang, Mi Ni Huang, Vincent Huang, Yi Huang, Wolfgang Huber, Thomas J. Hudson, Michael Hummel, Jillian A. Hung, David Huntsman, Ted R. Hupp, Jason Huse, Matthew R. Huska, Barbara Hutter, Carolyn M. Hutter, Daniel Hübschmann, Christine A. Iacobuzio-Donahue, Charles David Imbusch, Marcin Imielinski, Seiya Imoto, William B. Isaacs, Keren Isaev, Shumpei Ishikawa, Murat Iskar, S. M. Ashiqul Islam, Michael Ittmann, Sinisa Ivkovic, Jose M. G. Izarzugaza, Jocelyne Jacquemier, Valerie Jakrot, Nigel B. Jamieson, Gun Ho Jang, Se Jin Jang, Joy C. Jayaseelan, Reyka Jayasinghe, Stuart R. Jefferys, Karine Jegalian, Jennifer L. Jennings, Seung-Hyup Jeon, Lara Jerman, Yuan Ji, Wei Jiao, Peter A. Johansson, Amber L. Johns, Jeremy Johns, Rory Johnson, Todd A. Johnson, Clemency Jolly, Yann Joly, Jon G. Jonasson, Corbin D. Jones, David R. Jones, David T. W. Jones, Nic Jones, Steven J. M. Jones, Jos Jonkers, Young Seok Ju, Hartmut Juhl, Jongsun Jung, Malene Juul, Randi Istrup Juul, Sissel Juul, Natalie Jäger, Rolf Kabbe, Andre Kahles, Abdullah Kahraman, Vera B. Kaiser, Hojabr Kakavand, Sangeetha Kalimuthu, Christof von Kalle, Koo Jeong Kang, Katalin Karaszi, Beth Karlan, Rosa Karlić, Dennis Karsch, Katayoon Kasaian, Karin S. Kassahn, Hitoshi Katai, Mamoru Kato, Hiroto Katoh, Yoshiiku Kawakami, Jonathan D. Kay, Stephen H. Kazakoff, Marat D. Kazanov, Maria Keays, Electron Kebebew, Richard F. Kefford, Manolis Kellis, James G. Kench, Catherine J. Kennedy, Jules N. A. Kerssemakers, David Khoo, Vincent Khoo, Narong Khuntikeo, Ekta Khurana, Helena Kilpinen, Hark Kyun Kim, Hyung-Lae Kim, Hyung-Yong Kim, Hyunghwan Kim, Jaegil Kim, Jihoon Kim, Jong K. Kim, Youngwook Kim, Tari A. King, Wolfram Klapper, Kortine Kleinheinz, Leszek J. Klimczak, Stian Knappskog, Michael Kneba, Bartha M. Knoppers, Youngil Koh, Jan Komorowski, Daisuke Komura, Mitsuhiro Komura, Gu Kong, Marcel Kool, Jan O. Korbel, Viktoriya Korchina, Andrey Korshunov, Michael Koscher, Roelof Koster, Zsofia Kote-Jarai, Antonios Koures, Milena Kovacevic, Barbara Kremeyer, Helene Kretzmer, Markus Kreuz, Savitri Krishnamurthy, Dieter Kube, Kiran Kumar, Pardeep Kumar, Sushant Kumar, Yogesh Kumar, Ritika Kundra, Kirsten Kübler, Ralf Küppers, Jesper Lagergren, Phillip H. Lai, Peter W. Laird, Sunil R. Lakhani, Christopher M. Lalansingh, Emilie Lalonde, Fabien C. Lamaze, Adam Lambert, Eric Lander, Pablo Landgraf, Luca Landoni, Anita Langerød, Andrés Lanzós, Denis Larsimont, Erik Larsson, Mark Lathrop, Loretta M. S. Lau, Chris Lawerenz, Rita T. Lawlor, Michael S. Lawrence, Alexander J. Lazar, Ana Mijalkovic Lazic, Xuan Le, Darlene Lee, Donghoon Lee, Eunjung Alice Lee, Hee Jin Lee, Jake June-Koo Lee, Jeong-Yeon Lee, Juhee Lee, Ming Ta Michael Lee, Henry Lee-Six, Kjong-Van Lehmann, Hans Lehrach, Dido Lenze, Conrad R. Leonard, Daniel A. Leongamornlert, Ignaty Leshchiner, Louis Letourneau, Ivica Letunic, Douglas A. Levine, Lora Lewis, Tim Ley, Chang Li, Constance H. Li, Haiyan Irene Li, Jun Li, Lin Li, Shantao Li, Siliang Li, Xiaobo Li, Xiaotong Li, Xinyue Li, Yilong Li, Han Liang, Sheng-Ben Liang, Peter Lichter, Pei Lin, Ziao Lin, W. M. Linehan, Ole Christian Lingjærde, Dongbing Liu, Eric Minwei Liu, Fei-Fei Fei Liu, Fenglin Liu, Jia Liu, Xingmin Liu, Julie Livingstone, Dimitri Livitz, Naomi Livni, Lucas Lochovsky, Markus Loeffler, Georgina V. Long, Armando Lopez-Guillermo, Shaoke Lou, David N. Louis, Laurence B. Lovat, Yiling Lu, Yong-Jie Lu, Youyong Lu, Claudio Luchini, Ilinca Lungu, Xuemei Luo, Hayley J. Luxton, Andy G. Lynch, Lisa Lype, Cristina López, Carlos López-Otín, Eric Z. Ma, Yussanne Ma, Gaetan MacGrogan, Shona MacRae, Geoff Macintyre, Tobias Madsen, Kazuhiro Maejima, Andrea Mafficini, Dennis T. Maglinte, Arindam Maitra, Partha P. Majumder, Luca Malcovati, Salem Malikic, Giuseppe Malleo, Graham J. Mann, Luisa Mantovani-Löffler, Kathleen Marchal, Giovanni Marchegiani, Elaine R. Mardis, Adam A. Margolin, Maximillian G. Marin, Florian Markowetz, Julia Markowski, Jeffrey Marks, Tomas Marques-Bonet, Marco A. Marra, Luke Marsden, John W. M. Martens, Sancha Martin, Jose I. Martin-Subero, Iñigo Martincorena, Alexander Martinez-Fundichely, Yosef E. Maruvka, R. Jay Mashl, Charlie E. Massie, Thomas J. Matthew, Lucy Matthews, Erik Mayer, Simon Mayes, Michael Mayo, Faridah Mbabaali, Karen McCune, Ultan McDermott, Patrick D. McGillivray, Michael D. McLellan, John D. McPherson, John R. McPherson, Treasa A. McPherson, Samuel R. Meier, Alice Meng, Shaowu Meng, Andrew Menzies, Neil D. Merrett, Sue Merson, Matthew Meyerson, William Meyerson, Piotr A. Mieczkowski, George L. Mihaiescu, Sanja Mijalkovic, Tom Mikkelsen, Michele Milella, Linda Mileshkin, Christopher A. Miller, David K. Miller, Jessica K. Miller, Gordon B. Mills, Ana Milovanovic, Sarah Minner, Marco Miotto, Gisela Mir Arnau, Lisa Mirabello, Chris Mitchell, Thomas J. Mitchell, Satoru Miyano, Naoki Miyoshi, Shinichi Mizuno, Fruzsina Molnár-Gábor, Malcolm J. Moore, Richard A. Moore, Sandro Morganella, Quaid D. Morris, Carl Morrison, Lisle E. Mose, Catherine D. Moser, Ferran Muiños, Loris Mularoni, Andrew J. Mungall, Karen Mungall, Elizabeth A. Musgrove, Ville Mustonen, David Mutch, Francesc Muyas, Donna M. Muzny, Alfonso Muñoz, Jerome Myers, Ola Myklebost, Peter Möller, Genta Nagae, Adnan M. Nagrial, Hardeep K. Nahal-Bose, Hitoshi Nakagama, Hidewaki Nakagawa, Hiromi Nakamura, Toru Nakamura, Kaoru Nakano, Tannistha Nandi, Jyoti Nangalia, Mia Nastic, Arcadi Navarro, Fabio C. P. Navarro, David E. Neal, Gerd Nettekoven, Felicity Newell, Steven J. Newhouse, Yulia Newton, Alvin Wei Tian Ng, Anthony Ng, Jonathan Nicholson, David Nicol, Yongzhan Nie, G. Petur Nielsen, Morten Muhlig Nielsen, Serena Nik-Zainal, Michael S. Noble, Katia Nones, Paul A. Northcott, Faiyaz Notta, Brian D. O’Connor, Peter O’Donnell, Maria O’Donovan, Sarah O’Meara, Brian Patrick O’Neill, J. Robert O’Neill, David Ocana, Angelica Ochoa, Layla Oesper, Christopher Ogden, Hideki Ohdan, Kazuhiro Ohi, Lucila Ohno-Machado, Karin A. Oien, Akinyemi I. Ojesina, Hidenori Ojima, Takuji Okusaka, Larsson Omberg, Choon Kiat Ong, Stephan Ossowski, German Ott, B. F. Francis Ouellette, Christine P’ng, Marta Paczkowska, Salvatore Paiella, Chawalit Pairojkul, Marina Pajic, Qiang Pan-Hammarström, Elli Papaemmanuil, Irene Papatheodorou, Nagarajan Paramasivam, Ji Wan Park, Joong-Won Park, Keunchil Park, Kiejung Park, Peter J. Park, Joel S. Parker, Simon L. Parsons, Harvey Pass, Danielle Pasternack, Alessandro Pastore, Ann-Marie Patch, Iris Pauporté, Antonio Pea, John V. Pearson, Chandra Sekhar Pedamallu, Jakob Skou Pedersen, Paolo Pederzoli, Martin Peifer, Nathan A. Pennell, Charles M. Perou, Marc D. Perry, Gloria M. Petersen, Myron Peto, Nicholas Petrelli, Robert Petryszak, Stefan M. Pfister, Mark Phillips, Oriol Pich, Hilda A. Pickett, Todd D. Pihl, Nischalan Pillay, Sarah Pinder, Mark Pinese, Andreia V. Pinho, Esa Pitkänen, Xavier Pivot, Elena Piñeiro-Yáñez, Laura Planko, Christoph Plass, Paz Polak, Tirso Pons, Irinel Popescu, Olga Potapova, Aparna Prasad, Shaun R. Preston, Manuel Prinz, Antonia L. Pritchard, Stephenie D. Prokopec, Elena Provenzano, Xose S. Puente, Sonia Puig, Montserrat Puiggròs, Sergio Pulido-Tamayo, Gulietta M. Pupo, Colin A. Purdie, Michael C. Quinn, Raquel Rabionet, Janet S. Rader, Bernhard Radlwimmer, Petar Radovic, Benjamin Raeder, Keiran M. Raine, Manasa Ramakrishna, Kamna Ramakrishnan, Suresh Ramalingam, Benjamin J. Raphael, W. Kimryn Rathmell, Tobias Rausch, Guido Reifenberger, Jüri Reimand, Jorge Reis-Filho, Victor Reuter, Iker Reyes-Salazar, Matthew A. Reyna, Sheila M. Reynolds, Esther Rheinbay, Yasser Riazalhosseini, Andrea L. Richardson, Julia Richter, Matthew Ringel, Markus Ringnér, Yasushi Rino, Karsten Rippe, Jeffrey Roach, Lewis R. Roberts, Nicola D. Roberts, Steven A. Roberts, A. Gordon Robertson, Alan J. Robertson, Javier Bartolomé Rodriguez, Bernardo Rodriguez-Martin, F. Germán Rodríguez-González, Michael H. A. Roehrl, Marius Rohde, Hirofumi Rokutan, Gilles Romieu, Ilse Rooman, Tom Roques, Daniel Rosebrock, Mara Rosenberg, Philip C. Rosenstiel, Andreas Rosenwald, Edward W. Rowe, Romina Royo, Steven G. Rozen, Yulia Rubanova, Mark A. Rubin, Carlota Rubio-Perez, Vasilisa A. Rudneva, Borislav C. Rusev, Andrea Ruzzenente, Gunnar Rätsch, Radhakrishnan Sabarinathan, Veronica Y. Sabelnykova, Sara Sadeghi, S. Cenk Sahinalp, Natalie Saini, Mihoko Saito-Adachi, Gordon Saksena, Adriana Salcedo, Roberto Salgado, Leonidas Salichos, Richard Sallari, Charles Saller, Roberto Salvia, Michelle Sam, Jaswinder S. Samra, Francisco Sanchez-Vega, Chris Sander, Grant Sanders, Rajiv Sarin, Iman Sarrafi, Aya Sasaki-Oku, Torill Sauer, Guido Sauter, Robyn P. M. Saw, Maria Scardoni, Christopher J. Scarlett, Aldo Scarpa, Ghislaine Scelo, Dirk Schadendorf, Jacqueline E. Schein, Markus B. Schilhabel, Matthias Schlesner, Thorsten Schlomm, Heather K. Schmidt, Sarah-Jane Schramm, Stefan Schreiber, Nikolaus Schultz, Steven E. Schumacher, Roland F. Schwarz, Richard A. Scolyer, David Scott, Ralph Scully, Raja Seethala, Ayellet V. Segre, Iris Selander, Colin A. Semple, Yasin Senbabaoglu, Subhajit Sengupta, Elisabetta Sereni, Stefano Serra, Dennis C. Sgroi, Mark Shackleton, Nimish C. Shah, Sagedeh Shahabi, Catherine A. Shang, Ping Shang, Ofer Shapira, Troy Shelton, Ciyue Shen, Hui Shen, Rebecca Shepherd, Ruian Shi, Yan Shi, Yu-Jia Shiah, Tatsuhiro Shibata, Juliann Shih, Eigo Shimizu, Kiyo Shimizu, Seung Jun Shin, Yuichi Shiraishi, Tal Shmaya, Ilya Shmulevich, Solomon I. Shorser, Charles Short, Raunak Shrestha, Suyash S. Shringarpure, Craig Shriver, Shimin Shuai, Nikos Sidiropoulos, Reiner Siebert, Anieta M. Sieuwerts, Lina Sieverling, Sabina Signoretti, Katarzyna O. Sikora, Michele Simbolo, Ronald Simon, Janae V. Simons, Jared T. Simpson, Peter T. Simpson, Samuel Singer, Nasa Sinnott-Armstrong, Payal Sipahimalani, Tara J. Skelly, Marcel Smid, Jaclyn Smith, Karen Smith-McCune, Nicholas D. Socci, Heidi J. Sofia, Matthew G. Soloway, Lei Song, Anil K. Sood, Sharmila Sothi, Christos Sotiriou, Cameron M. Soulette, Paul N. Span, Paul T. Spellman, Nicola Sperandio, Andrew J. Spillane, Oliver Spiro, Jonathan Spring, Johan Staaf, Peter F. Stadler, Peter Staib, Stefan G. Stark, Lucy Stebbings, Ólafur Andri Stefánsson, Oliver Stegle, Lincoln D. Stein, Alasdair Stenhouse, Chip Stewart, Stephan Stilgenbauer, Miranda D. Stobbe, Michael R. Stratton, Jonathan R. Stretch, Adam J. Struck, Joshua M. Stuart, Henk G. Stunnenberg, Hong Su, Xiaoping Su, Ren X. Sun, Stephanie Sungalee, Hana Susak, Akihiro Suzuki, Fred Sweep, Monika Szczepanowski, Holger Sültmann, Takashi Yugawa, Angela Tam, David Tamborero, Benita Kiat Tee Tan, Donghui Tan, Patrick Tan, Hiroko Tanaka, Hirokazu Taniguchi, Tomas J. Tanskanen, Maxime Tarabichi, Roy Tarnuzzer, Patrick Tarpey, Morgan L. Taschuk, Kenji Tatsuno, Simon Tavaré, Darrin F. Taylor, Amaro Taylor-Weiner, Jon W. Teague, Bin Tean Teh, Varsha Tembe, Javier Temes, Kevin Thai, Sarah P. Thayer, Nina Thiessen, Gilles Thomas, Sarah Thomas, Alan Thompson, Alastair M. Thompson, John F. F. Thompson, R. Houston Thompson, Heather Thorne, Leigh B. Thorne, Adrian Thorogood, Grace Tiao, Nebojsa Tijanic, Lee E. Timms, Roberto Tirabosco, Marta Tojo, Stefania Tommasi, Christopher W. Toon, Umut H. Toprak, David Torrents, Giampaolo Tortora, Jörg Tost, Yasushi Totoki, David Townend, Nadia Traficante, Isabelle Treilleux, Jean-Rémi Trotta, Lorenz H. P. Trümper, Ming Tsao, Tatsuhiko Tsunoda, Jose M. C. Tubio, Olga Tucker, Richard Turkington, Daniel J. Turner, Andrew Tutt, Masaki Ueno, Naoto T. Ueno, Christopher Umbricht, Husen M. Umer, Timothy J. Underwood, Lara Urban, Tomoko Urushidate, Tetsuo Ushiku, Liis Uusküla-Reimand, Alfonso Valencia, David J. Van Den Berg, Steven Van Laere, Peter Van Loo, Erwin G. Van Meir, Gert G. Van den Eynden, Theodorus Van der Kwast, Naveen Vasudev, Miguel Vazquez, Ravikiran Vedururu, Umadevi Veluvolu, Shankar Vembu, Lieven P. C. Verbeke, Peter Vermeulen, Clare Verrill, Alain Viari, David Vicente, Caterina Vicentini, K. VijayRaghavan, Juris Viksna, Ricardo E. Vilain, Izar Villasante, Anne Vincent-Salomon, Tapio Visakorpi, Douglas Voet, Paresh Vyas, Ignacio Vázquez-García, Nick M. Waddell, Nicola Waddell, Claes Wadelius, Lina Wadi, Rabea Wagener, Jeremiah A. Wala, Jian Wang, Jiayin Wang, Linghua Wang, Qi Wang, Wenyi Wang, Yumeng Wang, Zhining Wang, Paul M. Waring, Hans-Jörg Warnatz, Jonathan Warrell, Anne Y. Warren, Sebastian M. Waszak, David C. Wedge, Dieter Weichenhan, Paul Weinberger, John N. Weinstein, Joachim Weischenfeldt, Daniel J. Weisenberger, Ian Welch, Michael C. Wendl, Johannes Werner, Justin P. Whalley, David A. Wheeler, Hayley C. Whitaker, Dennis Wigle, Matthew D. Wilkerson, Ashley Williams, James S. Wilmott, Gavin W. Wilson, Julie M. Wilson, Richard K. Wilson, Boris Winterhoff, Jeffrey A. Wintersinger, Maciej Wiznerowicz, Stephan Wolf, Bernice H. Wong, Tina Wong, Winghing Wong, Youngchoon Woo, Scott Wood, Bradly G. Wouters, Adam J. Wright, Derek W. Wright, Mark H. Wright, Chin-Lee Wu, Dai-Ying Wu, Guanming Wu, Jianmin Wu, Kui Wu, Yang Wu, Zhenggang Wu, Liu Xi, Tian Xia, Qian Xiang, Xiao Xiao, Rui Xing, Heng Xiong, Qinying Xu, Yanxun Xu, Hong Xue, Shinichi Yachida, Sergei Yakneen, Rui Yamaguchi, Takafumi N. Yamaguchi, Masakazu Yamamoto, Shogo Yamamoto, Hiroki Yamaue, Fan Yang, Huanming Yang, Jean Y. Yang, Liming Yang, Lixing Yang, Shanlin Yang, Tsun-Po Yang, Yang Yang, Xiaotong Yao, Marie-Laure Yaspo, Lucy Yates, Christina Yau, Chen Ye, Kai Ye, Venkata D. Yellapantula, Christopher J. Yoon, Sung-Soo Yoon, Fouad Yousif, Jun Yu, Kaixian Yu, Willie Yu, Yingyan Yu, Ke Yuan, Yuan Yuan, Denis Yuen, Christina K. Yung, Olga Zaikova, Jorge Zamora, Marc Zapatka, Jean C. Zenklusen, Thorsten Zenz, Nikolajs Zeps, Cheng-Zhong Zhang, Fan Zhang, Hailei Zhang, Hongwei Zhang, Hongxin Zhang, Jiashan Zhang, Jing Zhang, Junjun Zhang, Xiuqing Zhang, Xuanping Zhang, Yan Zhang, Zemin Zhang, Zhongming Zhao, Liangtao Zheng, Xiuqing Zheng, Wanding Zhou, Yong Zhou, Bin Zhu, Hongtu Zhu, Jingchun Zhu, Shida Zhu, Lihua Zou, Xueqing Zou, Anna deFazio, Nicholas van As, Carolien H. M. van Deurzen, Marc J. van de Vijver, L. van’t Veer, Christian von Mering

**Affiliations:** 1 Division of Molecular Genetics, German Cancer Research Center (DKFZ), Heidelberg, Germany; 2 Informatics and Bio-computing Program, Ontario Institute for Cancer Research, Toronto, Ontario Canada; 3 Norwich Medical School, University of East Anglia, Norwich, UK; 4 Earlham Institute, Norwich, UK; 5 Heinrich-Pette-Institute, Leibniz Institute for Experimental Virology, Hamburg, Germany; 6 German Center for Infection Research (DZIF), Partner Site Hamburg-Borstel-Lübeck-Riems, Hamburg, Germany; 7 Bioinformatics Core, University Medical Center Hamburg-Eppendorf, Hamburg, Germany; 8 Bioinformatics Group, Department of Computer Science, University College London, London, UK; 9 Biomedical Data Science Laboratory, Francis Crick Institute, London, UK; 10 National Center for Tumor Diseases (NCT) Heidelberg, Heidelberg, Germany; 11 Division of Cancer Genome Research, German Cancer Research Center (DKFZ), Heidelberg, Germany; 12 German Cancer Consortium (DKTK), Heidelberg, Germany; 13 Department of Pathology and Molecular Pathology, University and University Hospital Zürich, Zurich, Switzerland; 15 Institute of Cancer Research, London, UK; 16 University of East Anglia, Norwich, UK; 17 Division of Theoretical Bioinformatics, German Cancer Research Center (DKFZ), Heidelberg, Germany; 18 Department of Bioinformatics and Functional Genomics, Institute of Pharmacy and Molecular Biotechnology, Heidelberg University and BioQuant Center, Heidelberg, Germany; 19 Center for Digital Health, Berlin Institute of Health and Charité Universitätsmedizin Berlin, Berlin, Germany; 20 Ontario Institute for Cancer Research, MaRS Centre, Toronto, Ontario Canada; 21 Department of Biochemistry and Molecular Medicine, University of Montreal, Montreal, Québec Canada; 23 RIKEN Center for Integrative Medical Sciences, Yokohama, Japan; 24 Department of Epidemiology, University of Alabama at Birmingham, Birmingham, AL USA; 25 HudsonAlpha Institute for Biotechnology, Huntsville, AL USA; 26 O’Neal Comprehensive Cancer Center, University of Alabama at Birmingham, Birmingham, AL USA; 27 Broad Institute of MIT and Harvard, Cambridge, MA USA; 28 Harvard Medical School, Boston, MA USA; 29 Department of Medical Oncology, Dana-Farber Cancer Institute, Boston, MA USA; 30 Bioinformatics and Omics Data Analytics, German Cancer Research Center (DKFZ), Heidelberg, Germany; 31 University of Texas MD Anderson Cancer Center, Houston, TX USA; 200 Applied Tumor Genomics Research Program, Research Programs Unit, University of Helsinki, Helsinki, Finland; 201 Wellcome Sanger Institute, Wellcome Genome Campus, Hinxton, UK; 202 Memorial Sloan Kettering Cancer Center, New York, NY USA; 203 Genome Science Division, Research Center for Advanced Science and Technology, University of Tokyo, Tokyo, Japan; 204 Department of Surgery, University of Chicago, Chicago, IL USA; 205 Department of Surgery, Division of Hepatobiliary and Pancreatic Surgery, School of Medicine, Keimyung University Dongsan Medical Center, Daegu, South Korea; 206 Department of Oncology, Gil Medical Center, Gachon University, Incheon, South Korea; 207 Hiroshima University, Hiroshima, Japan; 208 Department of Bioinformatics and Computational Biology, The University of Texas MD Anderson Cancer Center, Houston, TX USA; 209 University of Texas MD Anderson Cancer Center, Houston, TX USA; 210 King Faisal Specialist Hospital and Research Centre, Al Maather, Riyadh, Saudi Arabia; 211 Bioinformatics Unit, Spanish National Cancer Research Centre (CNIO), Madrid, Spain; 212 Bioinformatics Core Facility, University Medical Center Hamburg, Hamburg, Germany; 213 Heinrich Pette Institute, Leibniz Institute for Experimental Virology, Hamburg, Germany; 214 Ontario Tumour Bank, Ontario Institute for Cancer Research, Toronto, ON Canada; 215 Department of Pathology, The University of Texas MD Anderson Cancer Center, Houston, TX USA; 216 Laboratory of Pathology, Center for Cancer Research, National Cancer Institute, Bethesda, MD USA; 217 Department of Cellular and Molecular Medicine and Department of Bioengineering, University of California San Diego, La Jolla, CA USA; 218 UC San Diego Moores Cancer Center, San Diego, CA USA; 219 Canada’s Michael Smith Genome Sciences Centre, BC Cancer, Vancouver, BC Canada; 220 Sir Peter MacCallum Department of Oncology, Peter MacCallum Cancer Centre, University of Melbourne, Melbourne, VIC Australia; 221 Centre for Research in Molecular Medicine and Chronic Diseases (CiMUS), Universidade de Santiago de Compostela, Santiago de Compostela, Spain; 222 Department of Zoology, Genetics and Physical Anthropology, (CiMUS), Universidade de Santiago de Compostela, Santiago de Compostela, Spain; 223 The Biomedical Research Centre (CINBIO), Universidade de Vigo, Vigo, Spain; 224 Royal National Orthopaedic Hospital - Bolsover, London, UK; 225 Department of Genomic Medicine, The University of Texas MD Anderson Cancer Center, Houston, TX USA; 226 Quantitative and Computational Biosciences Graduate Program, Baylor College of Medicine, Houston, TX USA; 227 The Jackson Laboratory for Genomic Medicine, Farmington, CT USA; 228 Genome Informatics Program, Ontario Institute for Cancer Research, Toronto, ON Canada; 229 Institute of Human Genetics, Christian-Albrechts-University, Kiel, Germany; 230 Institute of Human Genetics, Ulm University and Ulm University Medical Center, Ulm, Germany; 231 Queensland Centre for Medical Genomics, Institute for Molecular Bioscience, University of Queensland, St. Lucia, Brisbane, QLD Australia; 232 Salford Royal NHS Foundation Trust, Salford, UK; 233 Department of Surgery, Pancreas Institute, University and Hospital Trust of Verona, Verona, Italy; 234 Molecular and Medical Genetics, OHSU Knight Cancer Institute, Oregon Health and Science University, Portland, OR USA; 235 Department of Molecular Oncology, BC Cancer Research Centre, Vancouver, BC Canada; 236 The McDonnell Genome Institute at Washington University, St. Louis, MO USA; 237 University College London, London, UK; 238 Division of Cancer Genomics, National Cancer Center Research Institute, National Cancer Center, Tokyo, Japan; 239 DLR Project Management Agency, Bonn, Germany; 240 Tokyo Women’s Medical University, Tokyo, Japan; 241 Center for Molecular Oncology, Memorial Sloan Kettering Cancer Center, New York, NY USA; 242 Los Alamos National Laboratory, Los Alamos, NM USA; 243 Department of Pathology, University Health Network, Toronto General Hospital, Toronto, ON Canada; 244 Nottingham University Hospitals NHS Trust, Nottingham, UK; 245 Epigenomics and Cancer Risk Factors, German Cancer Research Center (DKFZ), Heidelberg, Germany; 246 Computational Biology Program, Ontario Institute for Cancer Research, Toronto, ON Canada; 247 Department of Molecular Genetics, University of Toronto, Toronto, ON Canada; 248 Vector Institute, Toronto, ON Canada; 249 Hematopathology Section, Institute of Pathology, Christian-Albrechts-University, Kiel, Germany; 250 Department of Pathology and Laboratory Medicine, School of Medicine, University of North Carolina at Chapel Hill, Chapel Hill, NC USA; 251 Department of Cancer Genetics, Institute for Cancer Research, Oslo University Hospital, The Norwegian Radium Hospital, Oslo, Norway; 252 Pathology, Hospital Clinic, Institut d’Investigacions Biomèdiques August Pi i Sunyer (IDIBAPS), University of Barcelona, Barcelona, Spain; 253 Department of Veterinary Medicine, Transmissible Cancer Group, University of Cambridge, Cambridge, UK; 254 Alvin J. Siteman Cancer Center, Washington University School of Medicine, St. Louis, MO USA; 255 Wolfson Wohl Cancer Research Centre, Institute of Cancer Sciences, University of Glasgow, Glasgow, UK; 256 Lineberger Comprehensive Cancer Center, University of North Carolina at Chapel Hill, Chapel Hill, NC USA; 257 Broad Institute of MIT and Harvard, Cambridge, MA USA; 258 Dana-Farber/Boston Children’s Cancer and Blood Disorders Center, Boston, MA USA; 259 Department of Pediatrics, Harvard Medical School, Boston, MA USA; 260 Leeds Institute of Medical Research @ St. James’s, University of Leeds, St. James’s University Hospital, Leeds, UK; 261 Department of Pathology and Diagnostics, University and Hospital Trust of Verona, Verona, Italy; 262 Department of Surgery, Princess Alexandra Hospital, Brisbane, QLD Australia; 263 Surgical Oncology Group, Diamantina Institute, University of Queensland, Brisbane, QLD Australia; 264 Department of Population and Quantitative Health Sciences, Case Western Reserve University School of Medicine, Cleveland, OH USA; 265 Research Health Analytics and Informatics, University Hospitals Cleveland Medical Center, Cleveland, OH USA; 266 Gloucester Royal Hospital, Gloucester, UK; 267 European Molecular Biology Laboratory, European Bioinformatics Institute (EMBL-EBI), Cambridge, UK; 268 Diagnostic Development, Ontario Institute for Cancer Research, Toronto, ON Canada; 269 Barcelona Supercomputing Center (BSC), Barcelona, Spain; 270 Arnie Charbonneau Cancer Institute, University of Calgary, Calgary, AB Canada; 271 Departments of Surgery and Oncology, University of Calgary, Calgary, AB Canada; 272 Department of Pathology, Oslo University Hospital, The Norwegian Radium Hospital, Oslo, Norway; 273 PanCuRx Translational Research Initiative, Ontario Institute for Cancer Research, Toronto, ON Canada; 274 Department of Oncology, Sidney Kimmel Comprehensive Cancer Center at Johns Hopkins University School of Medicine, Baltimore, MD USA; 275 University Hospital Southampton NHS Foundation Trust, Southampton, UK; 276 Royal Stoke University Hospital, Stoke-on-Trent, UK; 277 Genome Sequence Informatics, Ontario Institute for Cancer Research, Toronto, ON Canada; 278 Human Longevity Inc, San Diego, CA USA; 279 Olivia Newton-John Cancer Research Institute, La Trobe University, Heidelberg, VIC Australia; 280 Computer Network Information Center, Chinese Academy of Sciences, Beijing, China; 281 Genome Canada, Ottawa, ON Canada; 282 CNAG-CRG, Centre for Genomic Regulation (CRG), Barcelona Institute of Science and Technology (BIST), Barcelona, Spain; 283 Universitat Pompeu Fabra (UPF), Barcelona, Spain; 284 Buck Institute for Research on Aging, Novato, CA USA; 285 Duke University Medical Center, Durham, NC USA; 286 Department of Human Genetics, Hannover Medical School, Hannover, Germany; 287 Center for Bioinformatics and Functional Genomics, Cedars-Sinai Medical Center, Los Angeles, CA USA; 288 Department of Biomedical Sciences, Cedars-Sinai Medical Center, Los Angeles, CA USA; 289 The Hebrew University Faculty of Medicine, Jerusalem, Israel; 290 Barts Cancer Institute, Barts and the London School of Medicine and Dentistry, Queen Mary University of London, London, UK; 291 Department of Computer Science, Bioinformatics Group, University of Leipzig, Leipzig, Germany; 292 Interdisciplinary Center for Bioinformatics, University of Leipzig, Leipzig, Germany; 293 Transcriptome Bioinformatics, LIFE Research Center for Civilization Diseases, University of Leipzig, Leipzig, Germany; 294 Department of Medical Oncology, Dana-Farber Cancer Institute, Boston, MA USA; 295 Department of Cancer Biology, Dana-Farber Cancer Institute, Boston, MA USA; 296 Harvard Medical School, Boston, MA USA; 297 USC Norris Comprehensive Cancer Center, University of Southern California, Los Angeles, CA USA; 298 Department of Diagnostics and Public Health, University and Hospital Trust of Verona, Verona, Italy; 299 Department of Mathematics, Aarhus University, Aarhus, Denmark; 300 Department of Molecular Medicine (MOMA), Aarhus University Hospital, Aarhus N, Denmark; 301 Instituto Carlos Slim de la Salud, Mexico City, Mexico; 302 Department of Medical Biophysics, University of Toronto, Toronto, ON Canada; 303 Cancer Division, Garvan Institute of Medical Research, Kinghorn Cancer Centre, University of New South Wales (UNSW Sydney), Sydney, NSW Australia; 304 South Western Sydney Clinical School, Faculty of Medicine, University of New South Wales (UNSW Sydney), Liverpool, NSW Australia; 305 West of Scotland Pancreatic Unit, Glasgow Royal Infirmary, Glasgow, UK; 306 Center for Digital Health, Berlin Institute of Health and Charitè - Universitätsmedizin Berlin, Berlin, Germany; 307 Heidelberg Center for Personalized Oncology (DKFZ-HIPO), German Cancer Research Center (DKFZ), Heidelberg, Germany; 308 The Preston Robert Tisch Brain Tumor Center, Duke University Medical Center, Durham, NC USA; 309 Massachusetts General Hospital, Boston, MA USA; 310 National Institute of Biomedical Genomics, Kalyani, West Bengal India; 311 Institute of Clinical Medicine and Institute of Oral Biology, University of Oslo, Oslo, Norway; 312 University of North Carolina at Chapel Hill, Chapel Hill, NC USA; 313 ARC-Net Centre for Applied Research on Cancer, University and Hospital Trust of Verona, Verona, Italy; 314 The Institute of Cancer Research, London, UK; 315 Centre for Computational Biology, Duke-NUS Medical School, Singapore, Singapore; 316 Programme in Cancer and Stem Cell Biology, Duke-NUS Medical School, Singapore, Singapore; 317 Division of Oncology and Pathology, Department of Clinical Sciences Lund, Lund University, Lund, Sweden; 318 Department of Pediatric Oncology, Hematology and Clinical Immunology, Heinrich-Heine-University, Düsseldorf, Germany; 319 Laboratory for Medical Science Mathematics, RIKEN Center for Integrative Medical Sciences, Yokohama, Japan; 320 RIKEN Center for Integrative Medical Sciences, Yokohama, Japan; 321 Department of Internal Medicine/Hematology, Friedrich-Ebert-Hospital, Neumünster, Germany; 322 Departments of Dermatology and Pathology, Yale University, New Haven, CT USA; 323 Centre for Genomic Regulation (CRG), The Barcelona Institute of Science and Technology, Barcelona, Spain; 324 Radcliffe Department of Medicine, University of Oxford, Oxford, UK; 325 Canadian Center for Computational Genomics, McGill University, Montreal, QC Canada; 326 Department of Human Genetics, McGill University, Montreal, QC Canada; 327 Department of Human Genetics, University of California Los Angeles, Los Angeles, CA USA; 328 Department of Pharmacology, University of Toronto, Toronto, ON Canada; 329 Faculty of Medicine and Health Technology, Tampere University and Tays Cancer Center, Tampere University Hospital, Tampere, Finland; 330 Haematology, Leeds Teaching Hospitals NHS Trust, Leeds, UK; 331 Translational Research and Innovation, Centre Léon Bérard, Lyon, France; 332 Fox Chase Cancer Center, Philadelphia, PA USA; 333 International Agency for Research on Cancer, World Health Organization, Lyon, France; 334 Earlham Institute, Norwich, UK; 335 Norwich Medical School, University of East Anglia, Norwich, UK; 336 Department of Molecular Biology, Faculty of Science, Radboud Institute for Molecular Life Sciences, Radboud University, Nijmegen, HB The Netherlands; 337 CRUK Manchester Institute and Centre, Manchester, UK; 338 Department of Radiation Oncology, University of Toronto, Toronto, ON Canada; 339 Division of Cancer Sciences, Manchester Cancer Research Centre, University of Manchester, Manchester, UK; 340 Radiation Medicine Program, Princess Margaret Cancer Centre, Toronto, ON Canada; 341 Department of Pathology, Brigham and Women’s Hospital, Harvard Medical School, Boston, MA USA; 342 Department of Surgery, Division of Thoracic Surgery, The Johns Hopkins University School of Medicine, Baltimore, MD USA; 343 Division of Molecular Pathology, The Netherlands Cancer Institute, Oncode Institute, Amsterdam, CX The Netherlands; 344 Department of Biomolecular Engineering, University of California Santa Cruz, Santa Cruz, CA USA; 345 UC Santa Cruz Genomics Institute, University of California Santa Cruz, Santa Cruz, CA USA; 346 Division of Applied Bioinformatics, German Cancer Research Center (DKFZ), Heidelberg, Germany; 347 German Cancer Consortium (DKTK), German Cancer Research Center (DKFZ), Heidelberg, Germany; 348 National Center for Tumor Diseases (NCT) Heidelberg, Heidelberg, Germany; 349 Center for Biological Sequence Analysis, Department of Bio and Health Informatics, Technical University of Denmark, Lyngby, Denmark; 350 Novo Nordisk Foundation Center for Protein Research, University of Copenhagen, Copenhagen, Denmark; 351 Institute for Molecular Bioscience, University of Queensland, St. Lucia, Brisbane, QLD Australia; 352 Biomedical Engineering, Oregon Health and Science University, Portland, OR USA; 353 Division of Theoretical Bioinformatics, German Cancer Research Center (DKFZ), Heidelberg, Germany; 354 Institute of Pharmacy and Molecular Biotechnology and BioQuant, Heidelberg University, Heidelberg, Germany; 355 Federal Ministry of Education and Research, Berlin, Germany; 356 Melanoma Institute Australia, University of Sydney, Sydney, NSW Australia; 357 Pediatric Hematology and Oncology, University Hospital Muenster, Muenster, Germany; 358 Department of Pathology, Johns Hopkins University School of Medicine, Baltimore, MD USA; 359 McKusick-Nathans Institute of Genetic Medicine, Sidney Kimmel Comprehensive Cancer Center at Johns Hopkins University School of Medicine, Baltimore, MD USA; 360 Foundation Medicine, Inc, Cambridge, MA USA; 361 Department of Biomedical Data Science, Stanford University School of Medicine, Stanford, CA USA; 362 Department of Genetics, Stanford University School of Medicine, Stanford, CA USA; 363 Bakar Computational Health Sciences Institute and Department of Pediatrics, University of California, San Francisco, CA USA; 364 Institute of Clinical Medicine, Faculty of Medicine, University of Oslo, Oslo, Norway; 365 National Cancer Institute, National Institutes of Health, Bethesda, MD USA; 366 Royal Marsden NHS Foundation Trust, London and Sutton, UK; 367 Genome Biology Unit, European Molecular Biology Laboratory (EMBL), Heidelberg, Germany; 368 Department of Oncology, University of Cambridge, Cambridge, UK; 369 Li Ka Shing Centre, Cancer Research UK Cambridge Institute, University of Cambridge, Cambridge, UK; 370 Institut Gustave Roussy, Villejuif, France; 371 Cambridge University Hospitals NHS Foundation Trust, Cambridge, UK; 372 Department of Haematology, University of Cambridge, Cambridge, UK; 373 Anatomia Patológica, Hospital Clinic, Institut d’Investigacions Biomèdiques August Pi i Sunyer (IDIBAPS), University of Barcelona, Barcelona, Spain; 374 Spanish Ministry of Science and Innovation, Madrid, Spain; 375 University of Michigan Comprehensive Cancer Center, Ann Arbor, MI USA; 376 Department for BioMedical Research, University of Bern, Bern, Switzerland; 377 Department of Medical Oncology, Inselspital, University Hospital and University of Bern, Bern, Switzerland; 378 Graduate School for Cellular and Biomedical Sciences, University of Bern, Bern, Switzerland; 379 University of Pavia, Pavia, Italy; 380 University of Alabama at Birmingham, Birmingham, AL USA; 381 UHN Program in BioSpecimen Sciences, Toronto General Hospital, Toronto, ON Canada; 382 Department of Urology, Icahn School of Medicine at Mount Sinai, New York, NY USA; 383 Centre for Law and Genetics, University of Tasmania, Sandy Bay Campus, Hobart, TAS Australia; 384 Faculty of Biosciences, Heidelberg University, Heidelberg, Germany; 385 Department of Biochemistry, Microbiology and Immunology, Faculty of Medicine, University of Ottawa, Ottawa, ON Canada; 386 Division of Anatomic Pathology, Mayo Clinic, Rochester, MN USA; 387 Division of Cancer Epidemiology and Genetics, National Cancer Institute, National Institutes of Health, Bethesda, MD USA; 388 Illawarra Shoalhaven Local Health District L3 Illawarra Cancer Care Centre, Wollongong Hospital, Wollongong, NSW Australia; 389 BioForA, French National Institute for Agriculture, Food, and Environment (INRAE), ONF, Orléans, France; 390 Department of Biostatistics, Bloomberg School of Public Health, Johns Hopkins University, Baltimore, MD USA; 391 University of California San Diego, San Diego, CA USA; 392 Division of Experimental Pathology, Mayo Clinic, Rochester, MN USA; 393 Centre for Cancer Research, The Westmead Institute for Medical Research, University of Sydney, Sydney, NSW Australia; 394 Department of Gynaecological Oncology, Westmead Hospital, Sydney, NSW Australia; 395 PDXen Biosystems Inc, Seoul, South Korea; 396 Korea Advanced Institute of Science and Technology, Daejeon, South Korea; 397 Electronics and Telecommunications Research Institute, Daejeon, South Korea; 398 Institut National du Cancer (INCA), Boulogne-Billancourt, France; 399 Department of Genetics, Informatics Institute, University of Alabama at Birmingham, Birmingham, AL USA; 400 Division of Medical Oncology, National Cancer Centre, Singapore, Singapore; 401 Medical Oncology, University and Hospital Trust of Verona, Verona, Italy; 402 Department of Pediatrics, University Hospital Schleswig-Holstein, Kiel, Germany; 403 Hepatobiliary/Pancreatic Surgical Oncology Program, University Health Network, Toronto, ON Canada; 404 School of Biological Sciences, University of Auckland, Auckland, New Zealand; 405 Department of Surgery, University of Melbourne, Parkville, VIC Australia; 406 The Murdoch Children’s Research Institute, Royal Children’s Hospital, Parkville, VIC Australia; 407 Walter and Eliza Hall Institute, Parkville, VIC Australia; 408 Vancouver Prostate Centre, Vancouver, Canada; 409 Lunenfeld-Tanenbaum Research Institute, Mount Sinai Hospital, Toronto, ON Canada; 410 University of East Anglia, Norwich, UK; 411 Norfolk and Norwich University Hospital NHS Trust, Norwich, UK; 412 Victorian Institute of Forensic Medicine, Southbank, VIC Australia; 413 Department of Biomedical Informatics, Harvard Medical School, Boston, MA USA; 414 Department of Chemistry, Centre for Molecular Science Informatics, University of Cambridge, Cambridge, UK; 415 Ludwig Center at Harvard Medical School, Boston, MA USA; 416 Human Genome Sequencing Center, Baylor College of Medicine, Houston, TX USA; 417 Peter MacCallum Cancer Centre, University of Melbourne, Melbourne, VIC Australia; 418 Physics Division, Optimization and Systems Biology Lab, Massachusetts General Hospital, Boston, MA USA; 419 Department of Medicine, Baylor College of Medicine, Houston, TX USA; 420 University of Cologne, Cologne, Germany; 421 International Genomics Consortium, Phoenix, AZ USA; 422 Genomics Research Program, Ontario Institute for Cancer Research, Toronto, ON Canada; 423 Barking Havering and Redbridge University Hospitals NHS Trust, Romford, UK; 424 Children’s Hospital at Westmead, University of Sydney, Sydney, NSW Australia; 425 Department of Medicine, Section of Endocrinology, University and Hospital Trust of Verona, Verona, Italy; 426 Computational Biology Center, Memorial Sloan Kettering Cancer Center, New York, NY USA; 427 Department of Biology, ETH Zurich, Zürich, Switzerland; 428 Department of Computer Science, ETH Zurich, Zurich, Switzerland; 429 SIB Swiss Institute of Bioinformatics, Lausanne, Switzerland; 430 Weill Cornell Medical College, New York, NY USA; 431 Academic Department of Medical Genetics, University of Cambridge, Addenbrooke’s Hospital, Cambridge, UK; 432 MRC Cancer Unit, University of Cambridge, Cambridge, UK; 433 Departments of Pediatrics and Genetics, University of North Carolina at Chapel Hill, Chapel Hill, NC USA; 434 Seven Bridges Genomics, Charlestown, MA USA; 435 Annai Systems, Inc, Carlsbad, CA USA; 436 Department of Pathology, General Hospital of Treviso, Department of Medicine, University of Padua, Treviso, Italy; 437 Department of Computational Biology, University of Lausanne, Lausanne, Switzerland; 438 Department of Genetic Medicine and Development, University of Geneva Medical School, Geneva, CH Switzerland; 439 Swiss Institute of Bioinformatics, University of Geneva, Geneva, CH Switzerland; 440 The Francis Crick Institute, London, UK; 441 University of Leuven, Leuven, Belgium; 442 Institute of Medical Genetics and Applied Genomics, University of Tübingen, Tübingen, Germany; 443 Computational and Systems Biology, Genome Institute of Singapore, Singapore, Singapore; 444 School of Computing, National University of Singapore, Singapore, Singapore; 445 Big Data Institute, Li Ka Shing Centre, University of Oxford, Oxford, UK; 446 Biomedical Data Science Laboratory, Francis Crick Institute, London, UK; 447 Bioinformatics Group, Department of Computer Science, University College London, London, UK; 448 The Edward S. Rogers Sr. Department of Electrical and Computer Engineering, University of Toronto, Toronto, ON Canada; 449 Breast Cancer Translational Research Laboratory JC Heuson, Institut Jules Bordet, Brussels, Belgium; 450 Department of Oncology, Laboratory for Translational Breast Cancer Research, KU Leuven, Leuven, Belgium; 451 Institute for Research in Biomedicine (IRB Barcelona), The Barcelona Institute of Science and Technology, Barcelona, Spain; 452 Research Program on Biomedical Informatics, Universitat Pompeu Fabra, Barcelona, Spain; 453 Division of Medical Oncology, Princess Margaret Cancer Centre, Toronto, ON Canada; 454 Department of Physiology and Biophysics, Weill Cornell Medicine, New York, NY USA; 455 Institute for Computational Biomedicine, Weill Cornell Medicine, New York, NY USA; 456 Department of Pathology, UPMC Shadyside, Pittsburgh, PA USA; 457 Independent Consultant, Wellesley, USA; 458 Department of Cell and Molecular Biology, Science for Life Laboratory, Uppsala University, Uppsala, Sweden; 459 Department of Medicine and Department of Genetics, Washington University School of Medicine, St. Louis, St. Louis, MO USA; 460 Hefei University of Technology, Anhui, China; 461 Translational Cancer Research Unit, GZA Hospitals St.-Augustinus, Center for Oncological Research, Faculty of Medicine and Health Sciences, University of Antwerp, Antwerp, Belgium; 462 Simon Fraser University, Burnaby, BC Canada; 463 University of Pennsylvania, Philadelphia, PA USA; 464 Faculty of Science and Technology, University of Vic—Central University of Catalonia (UVic-UCC), Vic, Spain; 465 The Wellcome Trust, London, UK; 466 The Hospital for Sick Children, Toronto, ON Canada; 467 Department of Pathology, Queen Elizabeth University Hospital, Glasgow, UK; 468 Department of Genetics and Computational Biology, QIMR Berghofer Medical Research Institute, Brisbane, QLD Australia; 469 Department of Oncology, Centre for Cancer Genetic Epidemiology, University of Cambridge, Cambridge, UK; 470 Department of Public Health and Primary Care, Centre for Cancer Genetic Epidemiology, University of Cambridge, Cambridge, UK; 471 Prostate Cancer Canada, Toronto, ON Canada; 472 University of Cambridge, Cambridge, UK; 473 Department of Laboratory Medicine, Translational Cancer Research, Lund University Cancer Center at Medicon Village, Lund University, Lund, Sweden; 474 Heidelberg University, Heidelberg, Germany; 475 New BIH Digital Health Center, Berlin Institute of Health (BIH) and Charité - Universitätsmedizin Berlin, Berlin, Germany; 476 CIBER Epidemiología y Salud Pública (CIBERESP), Madrid, Spain; 477 Research Group on Statistics, Econometrics and Health (GRECS), UdG, Barcelona, Spain; 478 Quantitative Genomics Laboratories (qGenomics), Barcelona, Spain; 479 Icelandic Cancer Registry, Icelandic Cancer Society, Reykjavik, Iceland; 480 State Key Laboratory of Cancer Biology, and Xijing Hospital of Digestive Diseases, Fourth Military Medical University, Shaanxi, China; 481 Department of Medicine (DIMED), Surgical Pathology Unit, University of Padua, Padua, Italy; 482 Rigshospitalet, Copenhagen, Denmark; 483 Center for Cancer Genomics, National Cancer Institute, National Institutes of Health, Bethesda, MD USA; 484 Department of Biochemistry and Molecular Medicine, University of Montreal, Montreal, QC Canada; 485 Australian Institute of Tropical Health and Medicine, James Cook University, Douglas, QLD Australia; 486 Department of Neuro-Oncology, Istituto Neurologico Besta, Milano, Italy; 487 Bioplatforms Australia, North Ryde, NSW Australia; 488 Department of Pathology (Research), University College London Cancer Institute, London, UK; 489 Department of Surgical Oncology, Princess Margaret Cancer Centre, Toronto, ON Canada; 490 Department of Medical Oncology, Josephine Nefkens Institute and Cancer Genomics Centre, Erasmus Medical Center, Rotterdam, CN The Netherlands; 491 The University of Queensland Thoracic Research Centre, The Prince Charles Hospital, Brisbane, QLD Australia; 492 CIBIO/InBIO - Research Center in Biodiversity and Genetic Resources, Universidade do Porto, Vairão, Portugal; 493 HCA Laboratories, London, UK; 494 University of Liverpool, Liverpool, UK; 495 The Azrieli Faculty of Medicine, Bar-Ilan University, Safed, Israel; 496 Department of Neurosurgery, University of Florida, Gainesville, FL USA; 497 Department of Pathology, Graduate School of Medicine, University of Tokyo, Tokyo, Japan; 498 University of Milano Bicocca, Monza, Italy; 499 BGI-Shenzhen, Shenzhen, China; 500 Department of Pathology, Oslo University Hospital Ulleval, Oslo, Norway; 501 Center for Biomedical Informatics, Harvard Medical School, Boston, MA USA; 502 Department Biochemistry and Molecular Biomedicine, University of Barcelona, Barcelona, Spain; 503 Office of Cancer Genomics, National Cancer Institute, National Institutes of Health, Bethesda, MD USA; 504 Cancer Epigenomics, German Cancer Research Center (DKFZ), Heidelberg, Germany; 505 Department of Cancer Biology, The University of Texas MD Anderson Cancer Center, Houston, TX USA; 506 Department of Surgical Oncology, The University of Texas MD Anderson Cancer Center, Houston, TX USA; 507 Department of Computer Science, Yale University, New Haven, CT USA; 508 Department of Molecular Biophysics and Biochemistry, Yale University, New Haven, CT USA; 509 Program in Computational Biology and Bioinformatics, Yale University, New Haven, CT USA; 510 Center for Cancer Research, Massachusetts General Hospital, Boston, MA USA; 511 Department of Pathology, Massachusetts General Hospital, Boston, MA USA; 512 Department of Pathology, Memorial Sloan Kettering Cancer Center, New York, NY USA; 513 Division of Gastroenterology and Hepatology, Mayo Clinic, Rochester, MN USA; 514 University of Sydney, Sydney, NSW Australia; 515 University of Oxford, Oxford, UK; 516 Department of Surgery, Academic Urology Group, University of Cambridge, Cambridge, UK; 517 Department of Medicine II, University of Würzburg, Wuerzburg, Germany; 518 Sylvester Comprehensive Cancer Center, University of Miami, Miami, FL USA; 519 Institut Hospital del Mar d’Investigacions Mèdiques (IMIM), Barcelona, Spain; 520 Genome Integrity and Structural Biology Laboratory, National Institute of Environmental Health Sciences (NIEHS), Durham, NC USA; 521 St. Thomas’s Hospital, London, UK; 522 Osaka International Cancer Center, Osaka, Japan; 523 Department of Pathology, Skåne University Hospital, Lund University, Lund, Sweden; 524 Department of Medical Oncology, Beatson West of Scotland Cancer Centre, Glasgow, UK; 525 National Human Genome Research Institute, National Institutes of Health, Bethesda, MD USA; 526 Centre for Cancer Research, Victorian Comprehensive Cancer Centre, University of Melbourne, Melbourne, VIC Australia; 527 Department of Medicine, Section of Hematology/Oncology, University of Chicago, Chicago, IL USA; 528 German Center for Infection Research (DZIF), Partner Site Hamburg-Borstel-Lübeck-Riems, Hamburg, Germany; 529 Bioinformatics Research Centre (BiRC), Aarhus University, Aarhus, Denmark; 530 Department of Biotechnology, Ministry of Science and Technology, Government of India, New Delhi, Delhi India; 531 National Cancer Centre Singapore, Singapore, Singapore; 532 Brandeis University, Waltham, MA USA; 533 Department of Urologic Sciences, University of British Columbia, Vancouver, BC Canada; 534 Department of Internal Medicine, Stanford University, Stanford, CA USA; 535 The University of Texas Health Science Center at Houston, Houston, TX USA; 536 Imperial College NHS Trust, Imperial College, London, INY UK; 537 Senckenberg Institute of Pathology, University of Frankfurt Medical School, Frankfurt, Germany; 538 Department of Medicine, Division of Biomedical Informatics, UC San Diego School of Medicine, San Diego, CA USA; 539 Center for Precision Health, School of Biomedical Informatics, The University of Texas Health Science Center, Houston, TX USA; 540 Oxford Nanopore Technologies, New York, NY USA; 541 Institute of Medical Science, University of Tokyo, Tokyo, Japan; 542 Howard Hughes Medical Institute, University of California Santa Cruz, Santa Cruz, CA USA; 543 Wakayama Medical University, Wakayama, Japan; 544 Department of Internal Medicine, Division of Medical Oncology, Lineberger Comprehensive Cancer Center, University of North Carolina at Chapel Hill, Chapel Hill, NC USA; 545 University of Tennessee Health Science Center for Cancer Research, Memphis, TN USA; 546 Department of Histopathology, Salford Royal NHS Foundation Trust, Salford, UK; 547 Faculty of Biology, Medicine and Health, University of Manchester, Manchester, UK; 548 BIOPIC, ICG and College of Life Sciences, Peking University, Beijing, China; 549 Peking-Tsinghua Center for Life Sciences, Peking University, Beijing, China; 550 Children’s Hospital of Philadelphia, Philadelphia, PA USA; 551 Department of Bioinformatics and Computational Biology and Department of Systems Biology, The University of Texas MD Anderson Cancer Center, Houston, TX USA; 552 Karolinska Institute, Stockholm, Sweden; 553 The Donnelly Centre, University of Toronto, Toronto, ON Canada; 554 Department of Medical Genetics, College of Medicine, Hallym University, Chuncheon, South Korea; 555 Department of Experimental and Health Sciences, Institute of Evolutionary Biology (UPF-CSIC), Universitat Pompeu Fabra, Barcelona, Spain; 556 Health Data Science Unit, University Clinics, Heidelberg, Germany; 557 Massachusetts General Hospital Center for Cancer Research, Charlestown, MA USA; 558 Hokkaido University, Sapporo, Japan; 559 Department of Pathology and Clinical Laboratory, National Cancer Center Hospital, Tokyo, Japan; 560 Department of Genetics, University of North Carolina at Chapel Hill, Chapel Hill, NC USA; 561 Computational Biology, Leibniz Institute on Aging - Fritz Lipmann Institute (FLI), Jena, Germany; 562 University of Melbourne Centre for Cancer Research, Melbourne, VIC Australia; 563 University of Nebraska Medical Center, Omaha, NE USA; 564 Syntekabio Inc, Daejeon, South Korea; 565 Department of Pathology, Academic Medical Center, Amsterdam, AZ The Netherlands; 566 China National GeneBank-Shenzhen, Shenzhen, China; 567 Division of Molecular Genetics, German Cancer Research Center (DKFZ), Heidelberg, Germany; 568 Division of Life Science and Applied Genomics Center, Hong Kong University of Science and Technology, Clear Water Bay, Hong Kong, China; 569 Icahn School of Medicine at Mount Sinai, New York, NY USA; 570 Geneplus-Shenzhen, Shenzhen, China; 571 School of Computer Science and Technology, Xi’an Jiaotong University, Xi’an, China; 572 AbbVie, North Chicago, IL USA; 573 Institute of Pathology, Charité – University Medicine Berlin, Berlin, Germany; 574 Centre for Translational and Applied Genomics, British Columbia Cancer Agency, Vancouver, BC Canada; 575 Edinburgh Royal Infirmary, Edinburgh, UK; 576 Berlin Institute for Medical Systems Biology, Max Delbrück Center for Molecular Medicine, Berlin, Germany; 577 Department of Pediatric Immunology, Hematology and Oncology, University Hospital, Heidelberg, Germany; 578 German Cancer Research Center (DKFZ), Heidelberg, Germany; 579 Heidelberg Institute for Stem Cell Technology and Experimental Medicine (HI-STEM), Heidelberg, Germany; 580 Institute for Computational Biomedicine, Weill Cornell Medical College, New York, NY USA; 581 New York Genome Center, New York, NY USA; 582 Department of Urology, James Buchanan Brady Urological Institute, Johns Hopkins University School of Medicine, Baltimore, MD USA; 583 Department of Preventive Medicine, Graduate School of Medicine, The University of Tokyo, Tokyo, Japan; 584 Department of Molecular and Cellular Biology, Baylor College of Medicine, Houston, TX USA; 585 Department of Pathology and Immunology, Baylor College of Medicine, Houston, TX USA; 586 Michael E. DeBakey Veterans Affairs Medical Center, Houston, TX USA; 587 Technical University of Denmark, Lyngby, Denmark; 588 Department of Pathology, College of Medicine, Hanyang University, Seoul, South Korea; 589 Academic Unit of Surgery, School of Medicine, College of Medical, Veterinary and Life Sciences, University of Glasgow, Glasgow Royal Infirmary, Glasgow, UK; 590 Department of Pathology, Asan Medical Center, College of Medicine, Ulsan University, Songpa-gu, Seoul South Korea; 591 Science Writer, Garrett Park, MD USA; 592 International Cancer Genome Consortium (ICGC)/ICGC Accelerating Research in Genomic Oncology (ARGO) Secretariat, Ontario Institute for Cancer Research, Toronto, ON Canada; 593 University of Ljubljana, Ljubljana, Slovenia; 594 Department of Public Health Sciences, University of Chicago, Chicago, IL USA; 595 Research Institute, NorthShore University HealthSystem, Evanston, IL USA; 596 Department for Biomedical Research, University of Bern, Bern, Switzerland; 597 Centre of Genomics and Policy, McGill University and Génome Québec Innovation Centre, Montreal, QC Canada; 598 Carolina Center for Genome Sciences, University of North Carolina at Chapel Hill, Chapel Hill, NC USA; 599 Hopp Children’s Cancer Center (KiTZ), Heidelberg, Germany; 600 Pediatric Glioma Research Group, German Cancer Research Center (DKFZ), Heidelberg, Germany; 601 Cancer Research UK, London, UK; 602 Indivumed GmbH, Hamburg, Germany; 603 Genome Integration Data Center, Syntekabio, Inc, Daejeon, South Korea; 604 University Hospital Zurich, Zurich, Switzerland; 605 Clinical Bioinformatics, Swiss Institute of Bioinformatics, Geneva, Switzerland; 606 Institute for Pathology and Molecular Pathology, University Hospital Zurich, Zurich, Switzerland; 607 Institute of Molecular Life Sciences, University of Zurich, Zurich, Switzerland; 608 MRC Human Genetics Unit, MRC IGMM, University of Edinburgh, Edinburgh, UK; 609 Women’s Cancer Program at the Samuel Oschin Comprehensive Cancer Institute, Cedars-Sinai Medical Center, Los Angeles, CA USA; 610 Department of Biology, Bioinformatics Group, Division of Molecular Biology, Faculty of Science, University of Zagreb, Zagreb, Croatia; 611 Department for Internal Medicine II, University Hospital Schleswig-Holstein, Kiel, Germany; 612 Genetics and Molecular Pathology, SA Pathology, Adelaide, SA Australia; 613 Department of Gastric Surgery, National Cancer Center Hospital, Tokyo, Japan; 614 Department of Bioinformatics, Division of Cancer Genomics, National Cancer Center Research Institute, Tokyo, Japan; 615 A.A. Kharkevich Institute of Information Transmission Problems, Moscow, Russia; 616 Oncology and Immunology, Dmitry Rogachev National Research Center of Pediatric Hematology, Moscow, Russia; 617 Skolkovo Institute of Science and Technology, Moscow, Russia; 618 Department of Surgery, The George Washington University, School of Medicine and Health Science, Washington, DC USA; 619 Endocrine Oncology Branch, Center for Cancer Research, National Cancer Institute, National Institutes of Health, Bethesda, MD USA; 620 Melanoma Institute Australia, Macquarie University, Sydney, NSW Australia; 621 MIT Computer Science and Artificial Intelligence Laboratory, Massachusetts Institute of Technology, Cambridge, MA USA; 622 Tissue Pathology and Diagnostic Oncology, Royal Prince Alfred Hospital, Sydney, NSW Australia; 623 Cholangiocarcinoma Screening and Care Program and Liver Fluke and Cholangiocarcinoma Research Centre, Faculty of Medicine, Khon Kaen University, Khon Kaen, Thailand; 624 Controlled Department and Institution, New York, NY USA; 625 Englander Institute for Precision Medicine, Weill Cornell Medicine, New York, NY USA; 626 National Cancer Center, Gyeonggi, South Korea; 627 Department of Biochemistry, College of Medicine, Ewha Womans University, Seoul, South Korea; 628 Health Sciences Department of Biomedical Informatics, University of California San Diego, La Jolla, CA USA; 629 Research Core Center, National Cancer Centre Korea, Goyang-si, South Korea; 630 Department of Health Sciences and Technology, Sungkyunkwan University School of Medicine, Seoul, South Korea; 631 Samsung Genome Institute, Seoul, South Korea; 632 Breast Oncology Program, Dana-Farber/Brigham and Women’s Cancer Center, Boston, MA USA; 633 Department of Surgery, Memorial Sloan Kettering Cancer Center, New York, NY USA; 634 Division of Breast Surgery, Brigham and Women’s Hospital, Boston, MA USA; 635 Integrative Bioinformatics Support Group, National Institute of Environmental Health Sciences (NIEHS), Durham, NC USA; 636 Department of Clinical Science, University of Bergen, Bergen, Norway; 637 Center For Medical Innovation, Seoul National University Hospital, Seoul, South Korea; 638 Department of Internal Medicine, Seoul National University Hospital, Seoul, South Korea; 639 Institute of Computer Science, Polish Academy of Sciences, Warsawa, Poland; 640 Functional and Structural Genomics, German Cancer Research Center (DKFZ), Heidelberg, Germany; 641 Laboratory of Translational Genomics, Division of Cancer Epidemiology and Genetics, National Cancer Institute, , National Institutes of Health, Bethesda, MD USA; 642 Institute for Medical Informatics Statistics and Epidemiology, University of Leipzig, Leipzig, Germany; 643 Morgan Welch Inflammatory Breast Cancer Research Program and Clinic, The University of Texas MD Anderson Cancer Center, Houston, TX USA; 644 Department of Hematology and Oncology, Georg-Augusts-University of Göttingen, Göttingen, Germany; 645 Institute of Cell Biology (Cancer Research), University of Duisburg-Essen, Essen, Germany; 646 King’s College London and Guy’s and St. Thomas’ NHS Foundation Trust, London, UK; 647 Center for Epigenetics, Van Andel Research Institute, Grand Rapids, MI USA; 648 The University of Queensland Centre for Clinical Research, Royal Brisbane and Women’s Hospital, Herston, QLD Australia; 649 Department of Pediatric Oncology and Hematology, University of Cologne, Cologne, Germany; 650 University of Düsseldorf, Düsseldorf, Germany; 651 Department of Pathology, Institut Jules Bordet, Brussels, Belgium; 652 Institute of Biomedicine, Sahlgrenska Academy at University of Gothenburg, Gothenburg, Sweden; 653 Children’s Medical Research Institute, Sydney, NSW Australia; 654 ILSbio, LLC Biobank, Chestertown, MD USA; 655 Division of Genetics and Genomics, Boston Children’s Hospital, Harvard Medical School, Boston, MA USA; 656 Institute for Bioengineering and Biopharmaceutical Research (IBBR), Hanyang University, Seoul, South Korea; 657 Department of Statistics, University of California Santa Cruz, Santa Cruz, CA USA; 658 National Genotyping Center, Institute of Biomedical Sciences, Academia Sinica, Taipei, Taiwan; 659 Department of Vertebrate Genomics/Otto Warburg Laboratory Gene Regulation and Systems Biology of Cancer, Max Planck Institute for Molecular Genetics, Berlin, Germany; 660 McGill University and Genome Quebec Innovation Centre, Montreal, QC Canada; 661 biobyte solutions GmbH, Heidelberg, Germany; 662 Gynecologic Oncology, NYU Laura and Isaac Perlmutter Cancer Center, New York University, New York, NY USA; 663 Division of Oncology, Stem Cell Biology Section, Washington University School of Medicine, St. Louis, MO USA; 664 Department of Systems Biology, The University of Texas MD Anderson Cancer Center, Houston, TX USA; 665 Harvard University, Cambridge, MA USA; 666 Urologic Oncology Branch, Center for Cancer Research, National Cancer Institute, National Institutes of Health, Bethesda, MD USA; 667 University of Oslo, Oslo, Norway; 668 University of Toronto, Toronto, ON Canada; 669 Peking University, Beijing, China; 670 School of Life Sciences, Peking University, Beijing, China; 671 Leidos Biomedical Research, Inc, McLean, VA USA; 672 Hematology, Hospital Clinic, Institut d’Investigacions Biomèdiques August Pi i Sunyer (IDIBAPS), University of Barcelona, Barcelona, Spain; 673 Second Military Medical University, Shanghai, China; 674 Chinese Cancer Genome Consortium, Shenzhen, China; 675 Department of Medical Oncology, Beijing Hospital, Beijing, China; 676 Laboratory of Molecular Oncology, Key Laboratory of Carcinogenesis and Translational Research (Ministry of Education), Peking University Cancer Hospital and Institute, Beijing, China; 677 School of Medicine/School of Mathematics and Statistics, University of St. Andrews, St, Andrews, Fife UK; 678 Institute for Systems Biology, Seattle, WA USA; 679 Department of Biochemistry and Molecular Biology, Faculty of Medicine, University Institute of Oncology-IUOPA, Oviedo, Spain; 680 Institut Bergonié, Bordeaux, France; 681 Cancer Unit, MRC University of Cambridge, Cambridge, UK; 682 Department of Pathology and Laboratory Medicine, Center for Personalized Medicine, Children’s Hospital Los Angeles, Los Angeles, CA USA; 683 John Curtin School of Medical Research, Canberra, ACT Australia; 684 MVZ Department of Oncology, PraxisClinic am Johannisplatz, Leipzig, Germany; 685 Department of Information Technology, Ghent University, Ghent, Belgium; 686 Department of Plant Biotechnology and Bioinformatics, Ghent University, Ghent, Belgium; 687 Institute for Genomic Medicine, Nationwide Children’s Hospital, Columbus, OH USA; 688 Computational Biology Program, School of Medicine, Oregon Health and Science University, Portland, OR USA; 689 Department of Surgery, Duke University, Durham, NC USA; 690 Institució Catalana de Recerca i Estudis Avançats (ICREA), Barcelona, Spain; 691 Institut Català de Paleontologia Miquel Crusafont, Universitat Autònoma de Barcelona, Barcelona, Spain; 692 University of Glasgow, Glasgow, UK; 693 Institut d’Investigacions Biomèdiques August Pi i Sunyer (IDIBAPS), Barcelona, Spain; 694 Division of Oncology, Washington University School of Medicine, St. Louis, MO USA; 695 Department of Surgery and Cancer, Imperial College, London, INY UK; 696 Applications Department, Oxford Nanopore Technologies, Oxford, UK; 697 Department of Obstetrics, Gynecology and Reproductive Services, University of California San Francisco, San Francisco, CA USA; 698 Department of Biochemistry and Molecular Medicine, University California at Davis, Sacramento, CA USA; 699 STTARR Innovation Facility, Princess Margaret Cancer Centre, Toronto, ON Canada; 700 Discipline of Surgery, Western Sydney University, Penrith, NSW Australia; 701 Yale School of Medicine, Yale University, New Haven, CT USA; 702 Department of Genetics, Lineberger Comprehensive Cancer Center, University of North Carolina at Chapel Hill, Chapel Hill, NC USA; 703 Departments of Neurology and Neurosurgery, Henry Ford Hospital, Detroit, MI USA; 704 Precision Oncology, OHSU Knight Cancer Institute, Oregon Health and Science University, Portland, OR USA; 705 Institute of Pathology, University Medical Center Hamburg-Eppendorf, Hamburg, Germany; 706 Department of Health Sciences, Faculty of Medical Sciences, Kyushu University, Fukuoka, Japan; 707 Heidelberg Academy of Sciences and Humanities, Heidelberg, Germany; 708 Department of Clinical Pathology, University of Melbourne, Melbourne, VIC, Australia; 709 Department of Pathology, Roswell Park Cancer Institute, Buffalo, NY USA; 710 Department of Computer Science, University of Helsinki, Helsinki, Finland; 711 Institute of Biotechnology, University of Helsinki, Helsinki, Finland; 712 Organismal and Evolutionary Biology Research Programme, University of Helsinki, Helsinki, Finland; 713 Department of Obstetrics and Gynecology, Division of Gynecologic Oncology, Washington University School of Medicine, St. Louis, MO USA; 714 Penrose St. Francis Health Services, Colorado Springs, CO USA; 715 Institute of Pathology, Ulm University and University Hospital of Ulm, Ulm, Germany; 716 National Cancer Center, Tokyo, Japan; 717 Genome Institute of Singapore, Singapore, Singapore; 718 32Program in Computational Biology and Bioinformatics, Yale University, New Haven, CT USA; 719 German Cancer Aid, Bonn, Germany; 720 Programme in Cancer and Stem Cell Biology, Centre for Computational Biology, Duke-NUS Medical School, Singapore, Singapore; 721 The Chinese University of Hong Kong, Shatin, NT, Hong Kong China; 722 Fourth Military Medical University, Shaanxi, China; 723 The University of Cambridge School of Clinical Medicine, Cambridge, UK; 724 St. Jude Children’s Research Hospital, Memphis, TN USA; 725 University Health Network, Princess Margaret Cancer Centre, Toronto, ON Canada; 726 Center for Biomolecular Science and Engineering, University of California Santa Cruz, Santa Cruz, CA USA; 727 Department of Medicine, University of Chicago, Chicago, IL USA; 728 Department of Neurology, Mayo Clinic, Rochester, MN USA; 729 Cambridge Oesophagogastric Centre, Cambridge University Hospitals NHS Foundation Trust, Cambridge, UK; 730 Department of Computer Science, Carleton College, Northfield, MN USA; 731 Institute of Cancer Sciences, College of Medical Veterinary and Life Sciences, University of Glasgow, Glasgow, UK; 732 Department of Epidemiology, University of Alabama at Birmingham, Birmingham, AL USA; 733 HudsonAlpha Institute for Biotechnology, Huntsville, AL USA; 734 O’Neal Comprehensive Cancer Center, University of Alabama at Birmingham, Birmingham, AL USA; 735 Department of Pathology, Keio University School of Medicine, Tokyo, Japan; 736 Department of Hepatobiliary and Pancreatic Oncology, National Cancer Center Hospital, Tokyo, Japan; 737 Sage Bionetworks, Seattle, WA USA; 738 Lymphoma Genomic Translational Research Laboratory, National Cancer Centre, Singapore, Singapore; 739 Department of Clinical Pathology, Robert-Bosch-Hospital, Stuttgart, Germany; 740 Department of Cell and Systems Biology, University of Toronto, Toronto, ON Canada; 741 Department of Biosciences and Nutrition, Karolinska Institutet, Stockholm, Sweden; 742 Center for Liver Cancer, Research Institute and Hospital, National Cancer Center, Gyeonggi, South Korea; 743 Division of Hematology-Oncology, Samsung Medical Center, Sungkyunkwan University School of Medicine, Seoul, South Korea; 744 Samsung Advanced Institute for Health Sciences and Technology, Sungkyunkwan University School of Medicine, Seoul, South Korea; 745 Cheonan Industry-Academic Collaboration Foundation, Sangmyung University, Cheonan, South Korea; 746 NYU Langone Medical Center, New York, NY USA; 747 Department of Hematology and Medical Oncology, Cleveland Clinic, Cleveland, OH USA; 748 Department of Radiation Oncology, University of California San Francisco, San Francisco, CA USA; 749 Department of Health Sciences Research, Mayo Clinic, Rochester, MN USA; 750 Helen F. Graham Cancer Center at Christiana Care Health Systems, Newark, DE USA; 751 Heidelberg University Hospital, Heidelberg, Germany; 752 CSRA Incorporated, Fairfax, VA USA; 753 Research Department of Pathology, University College London Cancer Institute, London, UK; 754 Department of Research Oncology, Guy’s Hospital, King’s Health Partners AHSC, King’s College London School of Medicine, London, UK; 755 Faculty of Medicine and Health Sciences, Macquarie University, Sydney, NSW Australia; 756 University Hospital of Minjoz, INSERM UMR 1098, Besançon, France; 757 Spanish National Cancer Research Centre, Madrid, Spain; 758 Center of Digestive Diseases and Liver Transplantation, Fundeni Clinical Institute, Bucharest, Romania; 759 Cureline, Inc, South San Francisco, CA USA; 760 St. Luke’s Cancer Centre, Royal Surrey County Hospital NHS Foundation Trust, Guildford, UK; 761 Cambridge Breast Unit, Addenbrooke’s Hospital, Cambridge University Hospital NHS Foundation Trust and NIHR Cambridge Biomedical Research Centre, Cambridge, UK; 762 East of Scotland Breast Service, Ninewells Hospital, Aberdeen, UK; 763 Department of Genetics, Microbiology and Statistics, University of Barcelona, IRSJD, IBUB, Barcelona, Spain; 764 Department of Obstetrics and Gynecology, Medical College of Wisconsin, Milwaukee, WI USA; 765 Hematology and Medical Oncology, Winship Cancer Institute of Emory University, Atlanta, GA USA; 766 Department of Computer Science, Princeton University, Princeton, NJ USA; 767 Vanderbilt Ingram Cancer Center, Vanderbilt University, Nashville, TN USA; 768 Ohio State University College of Medicine and Arthur G. James Comprehensive Cancer Center, Columbus, OH USA; 769 Department of Surgery, Yokohama City University Graduate School of Medicine, Kanagawa, Japan; 770 Division of Chromatin Networks, German Cancer Research Center (DKFZ) and BioQuant, Heidelberg, Germany; 771 Research Computing Center, University of North Carolina at Chapel Hill, Chapel Hill, NC USA; 772 School of Molecular Biosciences and Center for Reproductive Biology, Washington State University, Pullman, WA USA; 773 Finsen Laboratory and Biotech Research and Innovation Centre (BRIC), University of Copenhagen, Copenhagen, Denmark; 774 Department of Laboratory Medicine and Pathobiology, University of Toronto, Toronto, ON Canada; 775 Department of Pathology, Human Oncology and Pathogenesis Program, Memorial Sloan Kettering Cancer Center, New York, NY USA; 776 University Hospital Giessen, Pediatric Hematology and Oncology, Giessen, Germany; 777 Oncologie Sénologie, ICM Institut Régional du Cancer, Montpellier, France; 778 Institute of Clinical Molecular Biology, Christian-Albrechts-University, Kiel, Germany; 779 Institute of Pathology, University of Wuerzburg, Wuerzburg, Germany; 780 Department of Urology, North Bristol NHS Trust, Bristol, UK; 781 SingHealth, Duke-NUS Institute of Precision Medicine, National Heart Centre Singapore, Singapore, Singapore; 782 Department of Computer Science, University of Toronto, Toronto, ON Canada; 783 Bern Center for Precision Medicine, University Hospital of Bern, University of Bern, Bern, Switzerland; 784 Englander Institute for Precision Medicine, Weill Cornell Medicine and New York Presbyterian Hospital, New York, NY USA; 785 Meyer Cancer Center, Weill Cornell Medicine, New York, NY USA; 786 Pathology and Laboratory, Weill Cornell Medical College, New York, NY USA; 787 Vall d’Hebron Institute of Oncology: VHIO, Barcelona, Spain; 788 General and Hepatobiliary-Biliary Surgery, Pancreas Institute, University and Hospital Trust of Verona, Verona, Italy; 789 National Centre for Biological Sciences, Tata Institute of Fundamental Research, Bangalore, India; 790 Indiana University, Bloomington, IN USA; 791 Department of Pathology, GZA-ZNA Hospitals, Antwerp, Belgium; 792 Analytical Biological Services, Inc, Wilmington, DE USA; 793 Sydney Medical School, University of Sydney, Sydney, NSW Australia; 794 cBio Center, Dana-Farber Cancer Institute, Harvard Medical School, Boston, MA USA; 795 Department of Cell Biology, Harvard Medical School, Boston, MA USA; 796 Advanced Centre for Treatment Research and Education in Cancer, Tata Memorial Centre, Navi Mumbai, Maharashtra India; 797 School of Environmental and Life Sciences, Faculty of Science, The University of Newcastle, Ourimbah, NSW Australia; 798 Department of Dermatology, University Hospital of Essen, Essen, Germany; 799 Bioinformatics and Omics Data Analytics, German Cancer Research Center (DKFZ), Heidelberg, Germany; 800 Department of Urology, Charité Universitätsmedizin Berlin, Berlin, Germany; 801 Martini-Clinic, Prostate Cancer Center, University Medical Center Hamburg-Eppendorf, Hamburg, Germany; 802 Department of General Internal Medicine, University of Kiel, Kiel, Germany; 803 German Cancer Consortium (DKTK), Partner site Berlin, Berlin, Germany; 804 Cancer Research Institute, Beth Israel Deaconess Medical Center, Boston, MA USA; 805 University of Pittsburgh, Pittsburgh, PA USA; 806 Department of Ophthalmology and Ocular Genomics Institute, Massachusetts Eye and Ear, Harvard Medical School, Boston, MA USA; 807 Center for Psychiatric Genetics, NorthShore University HealthSystem, Evanston, IL USA; 808 Van Andel Research Institute, Grand Rapids, MI USA; 809 Laboratory of Molecular Medicine, Human Genome Center, Institute of Medical Science, University of Tokyo, Tokyo, Japan; 810 Japan Agency for Medical Research and Development, Tokyo, Japan; 811 Korea University, Seoul, South Korea; 812 Murtha Cancer Center, Walter Reed National Military Medical Center, Bethesda, MD USA; 813 Human Genetics, University of Kiel, Kiel, Germany; 814 Department of Oncologic Pathology, Dana-Farber Cancer Institute, Harvard Medical School, Boston, MA USA; 815 Oregon Health and Science University, Portland, OR USA; 816 Center for RNA Interference and Noncoding RNA, The University of Texas MD Anderson Cancer Center, Houston, TX USA; 817 Department of Experimental Therapeutics, The University of Texas MD Anderson Cancer Center, Houston, TX USA; 818 Department of Gynecologic Oncology and Reproductive Medicine, The University of Texas MD Anderson Cancer Center, Houston, TX USA; 819 University Hospitals Coventry and Warwickshire NHS Trust, Coventry, UK; 820 Department of Radiation Oncology, Radboud University Nijmegen Medical Centre, Nijmegen, GA The Netherlands; 821 Institute for Genomics and Systems Biology, University of Chicago, Chicago, IL USA; 822 Clinic for Hematology and Oncology, St.-Antonius-Hospital, Eschweiler, Germany; 823 Computational and Systems Biology Program, Memorial Sloan Kettering Cancer Center, New York, NY USA; 824 University of Iceland, Reykjavik, Iceland; 825 Division of Computational Genomics and Systems Genetics, German Cancer Research Center (DKFZ), Heidelberg, Germany; 826 Dundee Cancer Centre, Ninewells Hospital, Dundee, UK; 827 Department for Internal Medicine III, University of Ulm and University Hospital of Ulm, Ulm, Germany; 828 Institut Curie, INSERM Unit 830, Paris, France; 829 Department of Gastroenterology and Hepatology, Yokohama City University Graduate School of Medicine, Kanagawa, Japan; 830 Department of Laboratory Medicine, Radboud University Nijmegen Medical Centre, Nijmegen, GA The Netherlands; 831 Division of Cancer Genome Research, German Cancer Research Center (DKFZ), Heidelberg, Germany; 832 Department of General Surgery, Singapore General Hospital, Singapore, Singapore; 833 Cancer Science Institute of Singapore, National University of Singapore, Singapore, Singapore; 834 Department of Medical and Clinical Genetics, Genome-Scale Biology Research Program, University of Helsinki, Helsinki, Finland; 835 East Anglian Medical Genetics Service, Cambridge University Hospitals NHS Foundation Trust, Cambridge, UK; 836 Irving Institute for Cancer Dynamics, Columbia University, New York, NY USA; 837 Institute of Molecular and Cell Biology, Singapore, Singapore; 838 Laboratory of Cancer Epigenome, Division of Medical Science, National Cancer Centre Singapore, Singapore, Singapore; 839 Universite Lyon, INCa-Synergie, Centre Léon Bérard, Lyon, France; 840 Department of Urology, Mayo Clinic, Rochester, MN USA; 841 Royal National Orthopaedic Hospital - Stanmore, Stanmore, Middlesex UK; 842 Department of Biochemistry, Genetics and Immunology, University of Vigo, Vigo, Spain; 843 Giovanni Paolo II / I.R.C.C.S. Cancer Institute, Bari, BA Italy; 844 Neuroblastoma Genomics, German Cancer Research Center (DKFZ), Heidelberg, Germany; 845 Fondazione Policlinico Universitario Gemelli IRCCS, Rome, Italy, Rome, Italy; 846 University of Verona, Verona, Italy; 847 Centre National de Génotypage, CEA - Institute de Génomique, Evry, France; 848 CAPHRI Research School, Maastricht University, Maastricht, ER The Netherlands; 849 Department of Biopathology, Centre Léon Bérard, Lyon, France; 850 Université Claude Bernard Lyon 1, Villeurbanne, France; 851 Core Research for Evolutional Science and Technology (CREST), JST, Tokyo, Japan; 852 Department of Biological Sciences, Laboratory for Medical Science Mathematics, Graduate School of Science, University of Tokyo, Yokohama, Japan; 853 Department of Medical Science Mathematics, Medical Research Institute, Tokyo Medical and Dental University (TMDU), Tokyo, Japan; 854 Cancer Ageing and Somatic Mutation Programme, Wellcome Sanger Institute, Hinxton, UK; 855 University Hospitals Birmingham NHS Foundation Trust, Birmingham, UK; 856 Centre for Cancer Research and Cell Biology, Queen’s University, Belfast, UK; 857 Breast Medical Oncology, The University of Texas MD Anderson Cancer Center, Houston, TX USA; 858 Department of Surgery, Johns Hopkins University School of Medicine, Baltimore, MD USA; 859 Department of Oncology-Pathology, Science for Life Laboratory, Karolinska Institute, Stockholm, Sweden; 860 School of Cancer Sciences, Faculty of Medicine, University of Southampton, Southampton, UK; 861 Department of Gene Technology, Tallinn University of Technology, Tallinn, Estonia; 862 Genetics and Genome Biology Program, SickKids Research Institute, The Hospital for Sick Children, Toronto, ON Canada; 863 Departments of Neurosurgery and Hematology and Medical Oncology, Winship Cancer Institute and School of Medicine, Emory University, Atlanta, GA USA; 864 Department of Clinical and Molecular Medicine, Faculty of Medicine and Health Sciences, Norwegian University of Science and Technology, Trondheim, Norway; 865 Argmix Consulting, North Vancouver, BC Canada; 866 Department of Information Technology, Ghent University, Interuniversitair Micro-Electronica Centrum (IMEC), Ghent, Belgium; 867 Nuffield Department of Surgical Sciences, John Radcliffe Hospital, University of Oxford, Oxford, UK; 868 Institute of Mathematics and Computer Science, University of Latvia, Riga, LV Latvia; 869 Discipline of Pathology, Sydney Medical School, University of Sydney, Sydney, NSW Australia; 870 Department of Applied Mathematics and Theoretical Physics, Centre for Mathematical Sciences, University of Cambridge, Cambridge, UK; 871 Department of Epidemiology and Biostatistics, Memorial Sloan Kettering Cancer Center, New York, NY USA; 872 Department of Statistics, Columbia University, New York, NY USA; 873 Department of Immunology, Genetics and Pathology, Science for Life Laboratory, Uppsala University, Uppsala, Sweden; 874 School of Electronic and Information Engineering, Xi’an Jiaotong University, Xi’an, China; 875 Department of Histopathology, Cambridge University Hospitals NHS Foundation Trust, Cambridge, UK; 876 Oxford NIHR Biomedical Research Centre, University of Oxford, Oxford, UK; 877 Georgia Regents University Cancer Center, Augusta, GA USA; 878 Wythenshawe Hospital, Manchester, UK; 879 Department of Genetics, Washington University School of Medicine, St.Louis, MO USA; 880 Department of Biological Oceanography, Leibniz Institute of Baltic Sea Research, Rostock, Germany; 881 Wellcome Centre for Human Genetics, University of Oxford, Oxford, UK; 882 Department of Molecular and Human Genetics, Baylor College of Medicine, Houston, TX USA; 883 Thoracic Oncology Laboratory, Mayo Clinic, Rochester, MN USA; 884 Institute for Genomic Medicine, Nationwide Children’s Hospital, Columbus, OH USA; 885 Department of Obstetrics and Gynecology, Division of Gynecologic Oncology, Mayo Clinic, Rochester, MN USA; 886 International Institute for Molecular Oncology, Poznań, Poland; 887 Poznan University of Medical Sciences, Poznań, Poland; 888 Genomics and Proteomics Core Facility High Throughput Sequencing Unit, German Cancer Research Center (DKFZ), Heidelberg, Germany; 889 NCCS-VARI Translational Research Laboratory, National Cancer Centre Singapore, Singapore, Singapore; 890 Edison Family Center for Genome Sciences and Systems Biology, Washington University, St. Louis, MO USA; 891 MRC-University of Glasgow Centre for Virus Research, Glasgow, UK; 892 Department of Medical Informatics and Clinical Epidemiology, Division of Bioinformatics and Computational Biology, OHSU Knight Cancer Institute, Oregon Health and Science University, Portland, OR USA; 893 School of Electronic Information and Communications, Huazhong University of Science and Technology, Wuhan, China; 894 Department of Applied Mathematics and Statistics, Johns Hopkins University, Baltimore, MD USA; 895 Department of Cancer Genome Informatics, Graduate School of Medicine, Osaka University, Osaka, Japan; 896 Institute of Computer Science, Heidelberg University, Heidelberg, Germany; 897 School of Mathematics and Statistics, University of Sydney, Sydney, NSW Australia; 898 Ben May Department for Cancer Research, University of Chicago, Chicago, IL USA; 899 Department of Human Genetics, University of Chicago, Chicago, IL USA; 900 Tri-Institutional PhD Program in Computational Biology and Medicine, Weill Cornell Medicine, New York, NY USA; 901 The First Affiliated Hospital, Xi’an Jiaotong University, Xi’an, China; 902 Department of Medicine and Therapeutics, The Chinese University of Hong Kong, Shatin, NT, Hong Kong China; 903 Department of Biostatistics, The University of Texas MD Anderson Cancer Center, Houston, TX USA; 904 Duke-NUS Medical School, Singapore, Singapore; 905 Department of Surgery, Ruijin Hospital, Shanghai Jiaotong University School of Medicine, Shanghai, China; 906 School of Computing Science, University of Glasgow, Glasgow, UK; 907 Division of Orthopaedic Surgery, Oslo University Hospital, Oslo, Norway; 908 Eastern Clinical School, Monash University, Melbourne, VIC Australia; 909 Epworth HealthCare, Richmond, VIC Australia; 910 Department of Biostatistics and Computational Biology, Dana-Farber Cancer Institute and Harvard Medical School, Boston, MA USA; 911 Department of Biomedical Informatics, College of Medicine, The Ohio State University, Columbus, OH USA; 912 The Ohio State University Comprehensive Cancer Center (OSUCCC – James), Columbus, OH USA; 913 The University of Texas School of Biomedical Informatics (SBMI) at Houston, Houston, TX USA; 914 Department of Biostatistics, University of North Carolina at Chapel Hill, Chapel Hill, NC USA; 915 Department of Biochemistry and Molecular Genetics, Feinberg School of Medicine, Northwestern University, Chicago, IL USA; 916 Faculty of Medicine and Health, University of Sydney, Sydney, NSW Australia; 917 Department of Pathology, Erasmus Medical Center Rotterdam, Rotterdam, GD The Netherlands; 918 Division of Molecular Carcinogenesis, The Netherlands Cancer Institute, Amsterdam, CX The Netherlands; 919 Institute of Molecular Life Sciences and Swiss Institute of Bioinformatics, University of Zurich, Zurich, Switzerland

**Keywords:** Cancer, Cancer

## Abstract

Here, as part of the Pan-Cancer Analysis of Whole Genomes (PCAWG) Consortium, for which whole-genome and—for a subset—whole-transcriptome sequencing data from 2,658 cancers across 38 tumor types was aggregated, we systematically investigated potential viral pathogens using a consensus approach that integrated three independent pipelines. Viruses were detected in 382 genome and 68 transcriptome datasets. We found a high prevalence of known tumor-associated viruses such as Epstein–Barr virus (EBV), hepatitis B virus (HBV) and human papilloma virus (HPV; for example, HPV16 or HPV18). The study revealed significant exclusivity of HPV and driver mutations in head-and-neck cancer and the association of HPV with APOBEC mutational signatures, which suggests that impaired antiviral defense is a driving force in cervical, bladder and head-and-neck carcinoma. For HBV, HPV16, HPV18 and adeno-associated virus-2 (AAV2), viral integration was associated with local variations in genomic copy numbers. Integrations at the *TERT* promoter were associated with high telomerase expression evidently activating this tumor-driving process. High levels of endogenous retrovirus (ERV1) expression were linked to a worse survival outcome in patients with kidney cancer.

## Abstract (web summary)

Viral pathogen load in cancer genomes is estimated through analysis of sequencing data from 2,656 tumors across 35 cancer types using multiple pathogen-detection pipelines, identifying viruses in 382 genomic and 68 transcriptome datasets.

## Main

The World Health Organization estimates that 15.4% of all cancers are attributable to infections and 9.9% are linked to viruses^1,2^. Cancers that are attributable to infections have a greater incidence than any individual type of cancer worldwide. Eleven pathogens have been classified as carcinogenic agents in humans by the International Agency for Research on Cancer (IARC)^3^. After *Helicobacter pylori* (associated with 770,000 cases worldwide), the four most prominent infection-related causes of cancer are estimated to be viral^2^: HPV^4^ (associated with 640,000 cases), HBV^5^ (420,000 cases), hepatitis C virus (HCV)^6^ (170,000 cases) and EBV^7^ (120,000 cases). It has been shown that viruses can contribute to the biology of multistep oncogenesis and are implicated in many of the hallmarks of cancer^8^. Notably, the discovery of links between infection and cancer types has provided actionable opportunities, such as the use of HPV vaccines as a preventive measure, to reduce the global impact of cancer. The following characteristics have been proposed to define human viruses that cause cancer through direct or indirect carcinogenesis^9^: (1) presence and persistence of viral DNA in tumor biopsies; (2) growth-promoting activity of viral genes in model systems; (3) dependence of a malignant phenotype on continuous viral oncogene expression or modification of host genes; and (4) epidemiological evidence that a virus infection represents a major risk for the development of cancer.

The worldwide efforts of comprehensive genome and whole-transcriptome analyses of tissue samples from patients with cancer have generated appropriate facilities for capturing information not only from human cells but also from other—potentially pathogenic—organisms or viruses that are present in the tissue. A comprehensive collection of whole-genome and whole-transcriptome data from cancer tissues has been generated within the International Cancer Genome Consortium (ICGC) project PCAWG^10^, providing a unique opportunity for a systematic search for tumor-associated viruses.

The PCAWG Consortium aggregated whole-genome sequencing (WGS) data from 2,658 cancers across 38 tumor types that have been generated by the ICGC and The Cancer Genome Atlas (TCGA) projects. These sequencing data were reanalyzed with standardized, high-accuracy pipelines to align to the human genome (build hs37d5) and identify germline variants and somatically acquired mutations^10^. The PCAWG working group ‘Pathogens’ analyzed the WGS and whole-transcriptome sequencing (RNA-sequencing (RNA-seq)) data of the PCAWG consensus cohort (2,656 donors). Focusing on viral pathogens, we applied three independently developed pathogen-detection pipelines ‘Computational Pathogen Sequence Identification’ (CaPSID)^11^, ‘Pathogen Discovery Pipeline’ (P-DiP) and ‘Searching for Pathogens’ (SEPATH) to generate a large compendium of viral associations across 38 cancer types. We extensively characterized the known and novel viral associations by integrating driver mutations, mutational signatures, gene expression profiles and patient survival data of the same set of tumors analyzed by the PCAWG Consortium.

## Results

### Identification of tumor-associated viruses

**Fig. 1:**
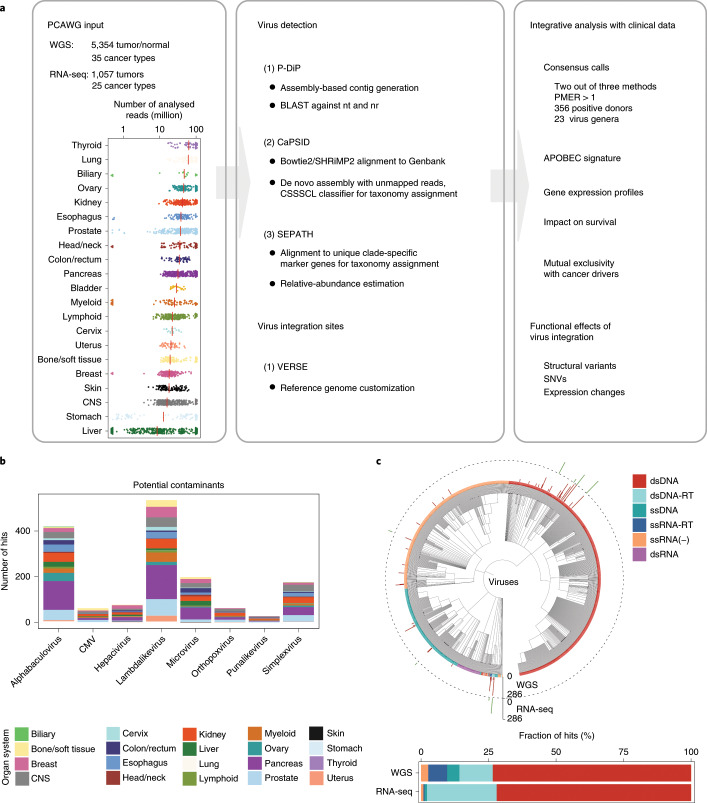
Overview, design and summary statistics. **a**, Workflow to identify and characterize viral sequences from the WGS and RNA sequencing of tumor and non-malignant samples. Viral hits were characterized in detail by using several clinical annotations and resources generated by PCAWG. The red line represents the median. CNS, central nervous system. **b**, Identified viral hits in contigs that showed higher viral reads PMER for artificial sequences such as vectors than for the virus. All viruses that occurred in at least 20 primary tumor samples in the same contig together with an artificial sequence are shown. **c**, Summary of the viral search space used in the analysis grouped by virus genome type. The number of virus-positive tumor samples is indicated in the outer rings (PMER log scale for WGS and RNA-seq data) as detected by any of the pipelines. Taxonomic relations between the viruses are indicated by the phylogenetic tree. dsDNA, double-stranded DNA; dsDNA-RT, double-stranded DNA with reverse transcriptase; dsRNA, double-stranded RNA; ssDNA, single-stranded DNA; ssRNA-RT: single-stranded RNA with reverse transcriptase; ssRNA, single-stranded RNA; dsRNA, double-stranded RNA. The fractions of hits in WGS and RNA-seq data are depicted as stacked bar graphs.

**Extended Data Fig. 1:**
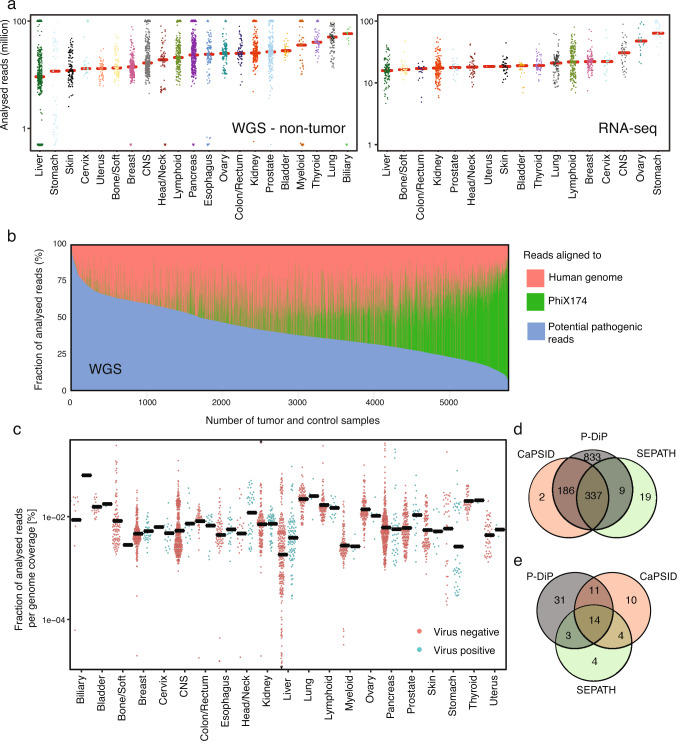
Statistics of analyzed reads from WGS and RNA-seq samples. **a**, Number of identified candidate pathogen reads used for WGS analysis in non-tumor samples and for RNA-seq analysis. Red line represents the median. **b**, Fraction of analysed reads mapped to phiX174 (green) and the human reference genome hg19 (red) and the rest labeled as potentially pathogenic reads (blue). **c**, Fraction of analysed reads per genome coverage separated for virus positive and negative tumor samples across organ systems. Thick black line represents the median. **d**, Search space overlap for genera across the three pipelines. **e**, Hit space overlap for genera across the three pipelines.

**Extended Data Fig. 2:**
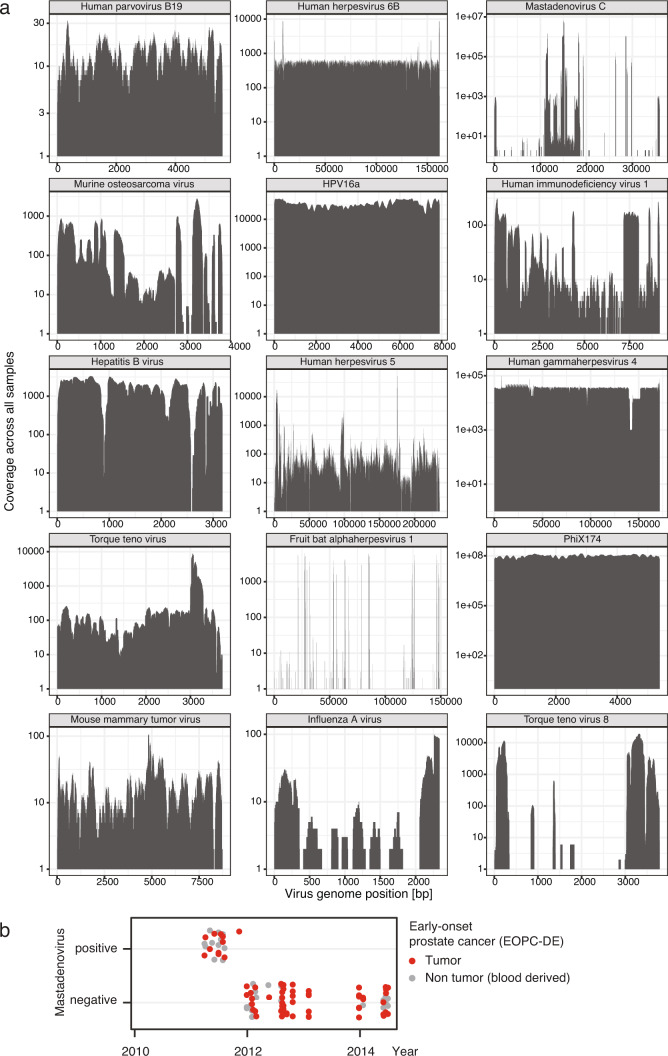
Genome coverage of mastadenovirus contamination detected in batches. **a**, Coverage of the virus genomes summarizing all mapped reads across all virus-positive tumor/normal samples. Alignment was done using BWA-mem. **b**, Mastadenovirus-positive samples ordered based on their sequencing date as years, indicating samples from early-onset prostate cancer (EOPC-DE) project across sequencing batches.

To identify the presence of viral sequences, we explored the WGS data of 5,354 tumor–normal samples across 38 cancer types, and 1,057 tumor RNA-seq data across 25 cancer types (Supplementary Tables 1, 2, 20). In total, 195.8 billion reads were considered for analysis, as they were not sufficiently aligned to the human reference genome in the PCAWG-generated alignment. The remaining reads ranged from 28,036 to 800 million reads per WGS and up to 120 million reads per RNA-seq tumor sample (Fig. 1a, Extended Data Fig. 1a–c). Viral sequences were detected and quantified independently by the three recently developed pathogen-discovery pipelines CaPSID, P-DiP and SEPATH. The estimated relative abundance of a virus was calculated as viral reads per million extracted reads (PMER) at the genus level to improve consistency between pipelines. To minimize the rate of false-positive hits in virus detection, we applied a strict threshold of PMER > 1 supported by at least three viral reads as suggested in previous studies^11,12^. Virus detection in a sample by at least two pipelines was considered to be a consensus hit. In total, 532 genera were considered for the extensive virus search in at least two of the pipelines (Extended Data Fig. 1d, Supplementary Table 18). Filtering of suspected viral laboratory contaminants was achieved through P-DiP, by examining each assembled contig of viral sequence segments for artificial, non-viral vector sequences and inspecting virus genome coverage across all positive samples (Extended Data Fig. 2a). The most frequent hits prone to suspected contamination were lambdavirus, alphabaculovirus, microvirus, simplexvirus, hepacivirus, cytomegalovirus (CMV), orthopoxvirus and punalikevirus; these were observed across many tumor types (Fig. 1b). For example, mastadenovirus showed an uneven genome coverage that could result from contaminating vector sequences. Therefore, we analyzed the virus detections across sequencing dates (Extended Data Fig. 2b) to assess any batch effect indicative of a contaminant; in mastadenovirus, we identified an association with sequencing date in early-onset prostate cancer regardless of tumor–normal state. We conclude that our mastadenovirus detections are due to a contamination that occurred across projects worldwide for which similar patterns could be identified.

**Fig. 2:**
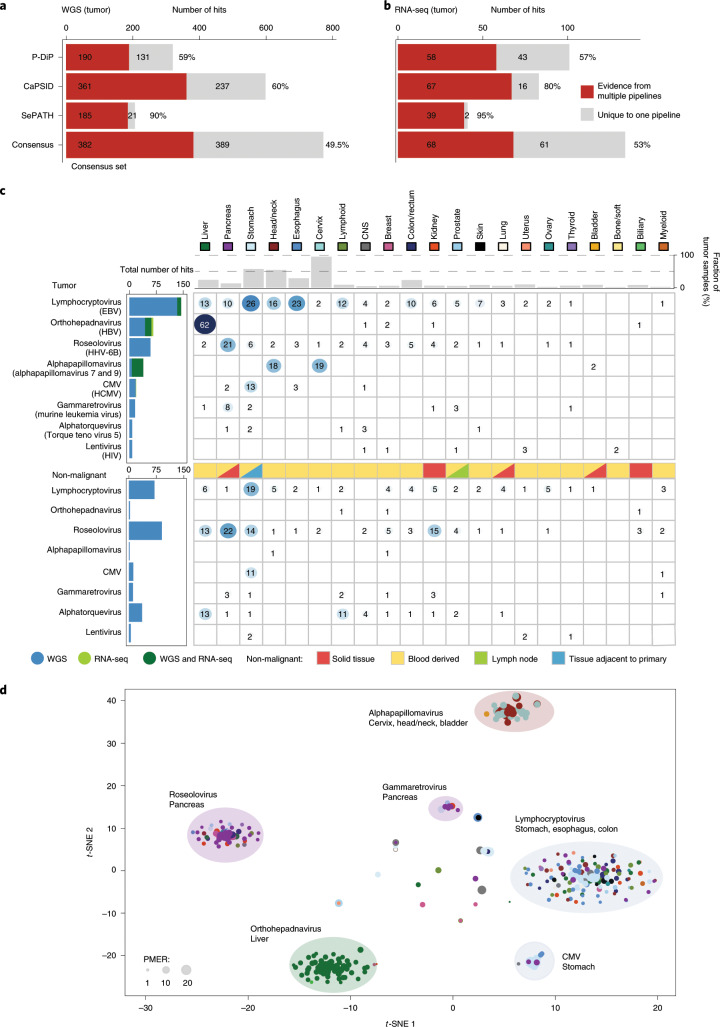
Consensus for detected viruses in WGS and RNA-seq data. Number of genus hits among tumor samples for the three independent pipelines and the consensus set defined by evidence from multiple pipelines. **a**, Analysis based on WGS. **b**, Analysis based on whole-transcriptome sequencing. **c**, Heat map showing the total number of viruses detected across various cancer entities. The sequencing data used for detection are indicated among the total number of hits (WGS, blue; RNA sequencing, green). The fraction of virus-positive samples is shown at the top and the type of non-malignant tissue used in the analysis is indicated if more than 15% of the analyzed samples are from a respective tissue type (solid tissue, lymph node, blood or adjacent to primary tumor). **d**, *t*-SNE clustering of the tumor samples based on PMER of their consensus virome profiles, using Pearson correlation as the distance metric. Major clusters are highlighted by indicating the strongest viral genus and the dominant tissue types that are positive in that cluster. Dot size represents the viral reads PMER.

**Extended Data Fig. 3:**
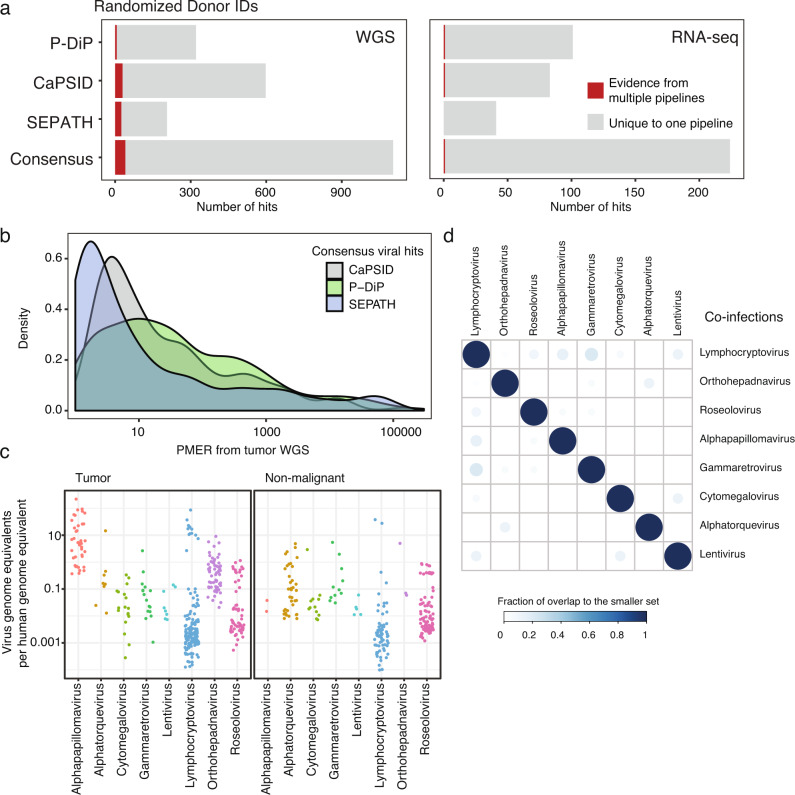
The distribution of PMER values for consensus hits across pathogen detection pipelines. **a**, Overlap calculated between three pipelines for the cases of shuffled viral hits randomized for their donor. **b**, PMER distribution of common viral hits detected by all three pipelines. **c**, Virus genome equivalents in relation to human tumor genome equivalents calculated for each sample positive for the virus. **d**, Co-infection of viruses detected in individual tumor samples. The fraction of overlap between two viruses were calculated as the number of shared samples divided by the smaller set.

We generally observed a strong overlap of the genera identified across pipelines (Extended Data Fig. 1e, Supplementary Tables 6, 7, 11). From the WGS dataset, we identified 321, 598 and 206 virus–tumor pairs using P-DiP, CaPSID and SEPATH, respectively (Fig. 2a; overlap after random permutation of detections, Extended Data Fig. 3a, Supplementary Tables 3–5). The number of hits derived from the RNA-seq dataset differed between the pipelines (virus–tumor pairs: 101 for P-DiP, 83 for CaPSID, 41 for SEPATH; Fig. 2b, Supplementary Tables 8–10). SEPATH, which used a *k*-mer approach, detected the lowest number of virus hits and was the least sensitive. Despite this, the identified viruses matched well with the consensus (DNA 90%, RNA 95%). P-DiP, which was based on an assembly and BLAST approach, detected more hits with 59% of the DNA and 54% of the RNA hits in the consensus set, whereas CaPSID, which was the most sensitive, implemented a two-step alignment process complemented with an assembly step and identified 60% (DNA) and 80% (RNA) hits within the consensus set. Although the majority of the virus hits from RNA-seq (*n* = 61 out of 68 consensus hits based on RNA-seq) overlapped with the WGS data, a lower fraction of detections from the WGS data were present in the RNA-seq data (*n* = 61 out of 168 of 382 consensus hits based on WGS with available RNA-seq data), emphasizing the importance of DNA sequencing for generating an unbiased catalog of tumor-associated viruses. This difference can also be attributed to the viral life cycle, as viral gene expression can be minimal during incubation or latent phases^13^. Contrasting virus-positive and virus-negative samples within each organ type shows that the organ system, as expected, has a significant influence, but virus positivity does not (*P* *<* 2 × 10^−16^, analysis of variance modeling of candidate reads that are dependent on organ system and virus positivity; Extended Data Fig. 1c). This indicates that virus-positive tumors were not detected owing to a higher number of candidate reads; this is consistent with the fact that the viral reads in most cases do not substantially contribute to the reads analyzed. In total, 86% of the sequence hits detected in WGS and RNA-seq data were found to be from double-stranded DNA viruses and double-stranded DNA viruses with reverse transcriptase (Fig. 1c, Supplementary Table 19). This could be attributed to (1) a higher frequency of tumor-associated viruses from these genome types^3^, (2) a larger sequencing dataset for WGS compared with RNA-seq, (3) a potential limitation of our analysis due to DNA and RNA extraction protocols that are less likely to include single-stranded DNA or RNA viruses or (4) the selection bias of tumor entities included in the PCAWG study (Fig. 1c).

### The virome landscape across 38 distinct tumor types

We used a consensus approach that resulted in a reliable set of 389 distinct virus–tumor pairs from WGS and RNA-seq data (Fig. 2a–d). Overall, 23 virus genera were detected across 356 patients with cancer (13%). The top-five most-prevalent viruses (lymphocryptovirus, orthohepadnavirus, roseolovirus, alphapapillomavirus and CMV) account for 85% of the consensus virus hits in tumors (*n* = 329 out of 389). Among these five prevalent virus genera, three have been well described in the literature as drivers of tumor initiation and progression^9^: (1) lymphocryptovirus (*n* = 145 samples (5.5%); for example, EBV) is the most common viral infection across a variety of tumor entities that mainly occur in the gastrointestinal tract and shows a much lower prevalence in the matched non-malignant control samples (*n* = 82 (3%); Fig. 2c); (2) orthohepadnavirus (*n* = 67 (2.5%); for example, HBV) is—as expected—the most frequent among liver cancer with HBV present in 62 of 330 donors (18.9%); and (3) alphapapillomavirus (discussed below). Lymphocryptovirus (*n* = 11), orthohepadnavirus (*n* = 18) and alphapapillomavirus (*n* = 32) were detected in both RNA-seq and DNA-sequencing data (Fig. 2c, left), of which alphapapillomavirus was the most frequent (32 out of 39 consensus hits). This is consistent with the constitutive expression of viral oncogenes in cancers associated with these viruses, a parameter that supports a direct role in carcinogenesis^9^. An in-depth analysis of the virus genome equivalents per human tumor genome equivalent, which considers genome sizes, coverage and tumor purity, showed overall low viral genome equivalents even for established tumor viruses (Extended Data Fig. 3c, Supplementary Table 12). Evidence of a mouse mammary tumor virus (MMTV, PMER = 3.4) was detected in one renal carcinoma sample and in none of the 214 analyzed breast cancer samples. Previous work has suggested that MMTV may have a role in breast cancer but our comprehensive search of viral sequences could not identify any MMTV-positive case in breast cancer that would support this claim.

**Extended Data Fig. 4:**
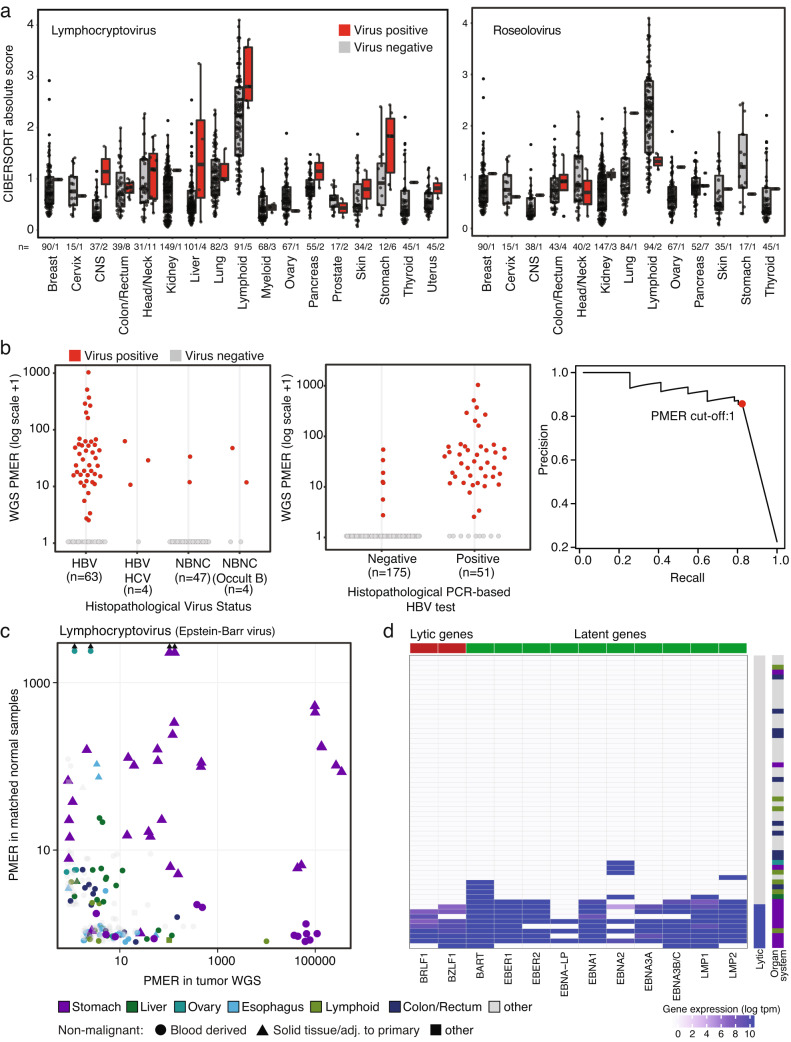
Specific findings for lymphocrypto- and roseolovirus, HBV and EBV. Overall contribution of immune cells across organ system in samples positive or negative for lymphocryptovirus and roseolovirus. Tukey boxplot indicates the median by the middle line and the 25–75th percentiles by the box. The whiskers were drawn up to the 1.5 interquartile range from the lower and upper quartile. **b**, Comparison of histopathologically detected HBV in liver cancer with the PMERs detected in WGS. Precision and recall of the PCR based HBV test versus the consensus calls from WGS data. Red dot indicates the PMER cut-off of 1. **c**, Relation of PMER for EBV detections in tumor and normal samples across organ system and normal tissue type. **d**, Epstein-Barr virus expression presenting lytic (red) and latent (green) genes across organ systems. Reads were counted after alignment with kallisto to the EBV reference transcriptome (see Methods).

Roseolovirus and alphatorquevirus show a higher number of hits in non-malignant control samples, which were mainly derived from blood cells (Fig. 2c). For example, we identified 59 patients as roseolovirus-positive (human herpesvirus (HHV)-6A, HHV-6B and HHV-7) in their tumors (pancreas, 6%; stomach, 8%; colon/rectum, 8.3%) and 90 patients positive in the non-malignant control samples. Considering the known cell tropism of roseolovirus for B and T cells^15^, we asked whether immune infiltration would be higher in roseolovirus-positive tumors. However, we could not identify a stronger contribution of immune cells in virus-positive tumor cases as estimated using CIBERSORT^14^ (false-discovery rate (FDR)-corrected *P* *>* 0.05 for pancreas; Extended Data Fig. 4a). Therefore, consistently with current knowledge (reviewed in ref. ^16^), we cannot confirm a link between roseolovirus and immune-cell content or tumor development. Furthermore, we could not identify actively transcribed viral genes for roseolovirus and alphatorquevirus at the transcriptome level. This is in agreement with the latent state of these viruses in blood mononuclear cells^15^, and their transmission through blood transfusions^17^. CMV was found, as expected^18^, after identification and removal of contaminations in both stomach tumors (*n* = 13) and the adjacent non-malignant tissue (*n* = 11). In line with a recent publication^19^, we could not detect CMV in the 294 tumors of the central nervous system (146 medulloblastomas, 89 pilocytic astrocytoma, 41 glioblastomas and 18 oligodendrogliomas) that were analyzed. Therefore, a previously debated role of this virus is not supported. Notably, we did not identify a significant enrichment of co-infection of multiple viruses in any tumor type (Extended Data Fig. 3d).

### Incidence of HBV

**Fig. 3:**
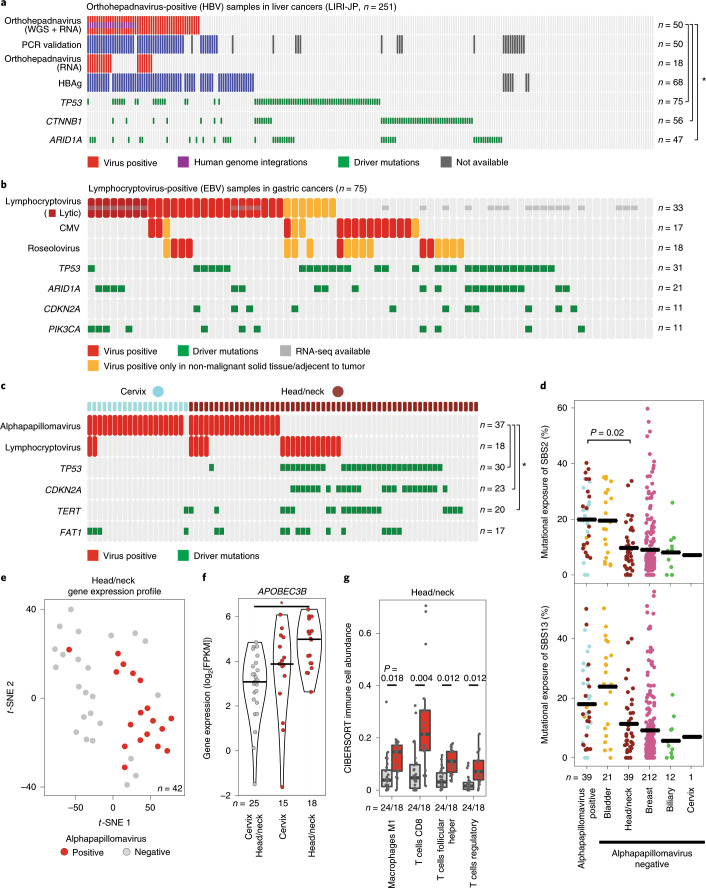
Virus-specific findings. **a**, HBV detections, validations and driver mutations in liver cancer. The asterisk indicates mutual exclusivity between HBV detection and somatic driver gene mutations. Red boxes represent virus-positive tumor samples, purple boxes show viral genomic integrations, green boxes indicate driver mutations and gray boxes represent missing data. **b**, Virus detections in gastric cancer samples, indication of virus phase (lytic/latent, dark red) and driver mutations (green). A yellow color indicates donors with virus-positive non-malignant samples. The gray box refers to samples with available RNA-seq data. **c**, Virus detections (red) and driver mutations (green) in cervix (blue) and head-and-neck cancer (brown). The asterisk indicates mutual exclusivity between alphapapillomavirus detections and somatic driver gene mutations. **d**, Alphapapillomavirus detection and exposures of mutational APOBEC signatures SBS2 and SBS13. Sample sizes are shown at the bottom. A two-sided Wilcoxon rank-sum test showed a significant difference (*P* = 0.02) of mutational signature exposure between virus-positive and virus-negative head-and-neck tumor samples. The black line indicates the median for each group. **e**, Gene expression analysis based a *t*-SNE map of head-and-neck cancer samples shows a distinct gene expression profile for virus-positive samples. Virus-positive and virus-negative samples are shown as red and gray dots, respectively. **f**, The violin plot of *APOBEC3B* gene expression for alphapapillomavirus-positive and alphapapillomavirus-negative samples in cervix and head-and-neck cancer (FDR-corrected two-sided Wilcoxon rank-sum test, *P* = 1.6 × 10^−4^). FPKM, fragments per kilobase of transcript per million mapped reads. The center line represents the median, and the upper and lower boundaries of the violin plot refer to the maximum and minimum values, respectively. **g**, Tumor-infiltrating immune cells as quantified by CIBERSORT using RNA-seq samples from patient with head-and-neck cancer. All four cell types showed significant enrichment of immune cells in virus-positive samples (FDR-corrected two-sided Wilcoxon rank-sum test, *n* = 24 virus negative versus 18 virus positive). Tukey box plots show the median (the middle line) and the 25–75th percentiles (the box); the whiskers show 1.5× the interquartile range from the lower and upper quartile.

HBV was most frequently detected in liver cancers (*n* = 62). Compared with the histopathological gold-standard HBV PCR test^20,21^ (*n* = 228), the WGS-based consensus detections had the same high specificity (96.1%) and a high sensitivity (84.0%), indicating that HBV detection using WGS is reliable (Fig. 3a, Extended Data Fig. 4b, Supplementary Table 13). Furthermore, five out of the seven cases that were positive using WGS but negative for HBV PCR showed positivity for HBAg, indicating that the WGS analysis has a high sensitivity. In summary, the precision (85.7%) and recall (84%) for the detection of HBV based on around 30-fold-coverage WGS data were comparable to those of targeted PCR. We confirmed a significant exclusivity between HBV infection and mutations in *CTNNB1*, *TP53* and *ARID1A* that was found in a larger liver cancer cohort analyzed by high-throughput sequencing (FDR-corrected *P* *=* 5.35 × 10^−6^, 0.0023 and 0.0023, respectively; DISCOVER^22^)^23^.

### Detection of EBV

EBV was detected in many different tumor entities and normal samples (Fig. 2c). When comparing the PMER of EBV in tumor and matched normal samples, we see a stronger contribution in matched normal samples from matched solid tissue or tissue adjacent to the tumor (Extended Data Fig. 4c). For samples that contained reads for EBV in WGS and with available RNA-seq data, the absolute score for immune cells based on CIBERSORT^14^ was not significantly different between virus-positive and virus-negative samples (FDR-corrected *P* > 0.05 for colon/rectum, head-and-neck, lymphoid and stomach; Extended Data Fig. 4a). In summary, there is no evidence that the detection of EBV is due to infiltrating immune cells. This indicates the presence of EBV in the respective organs. On the basis of the expression data available for the tumor samples, we identified viral transcripts of the latent as well as lytic phase of the viral life cycle (Fig. 3b, Extended Data Fig. 4d, Supplementary Table 13). Eight of the nine tumors that expressed lytic EBV transcripts were from stomach cancers, confirming the active contribution of EBV to gastric cancer^24^.

### Identification of alphapapillomaviruses

Alphapapillomaviruses were mainly detected in head-and-neck cancers (*n* = 18 out of 57), cervical cancers (*n* = 19 out of 20) and in two bladder cancer cases out of 23, in agreement with previous studies^4,25,26^. There is also supporting evidence for 32 out of 39 alphapapillomavirus hits in the whole-transcriptome data (Fig. 2c). We observed only one HPV subtype per tumor according to the P-DiP results and HPV16 was the dominant type in cervical (*n* = 11) and head-and-neck (*n* = 15) tumors, followed by HPV18, which was present in only cervical cancer (*n* = 6). As reported previously^27^, HPV33 was identified in head-and-neck (*n* = 3) and cervical (*n* = 1) tumors. Different HPV variants, type 6 and 45, were detected in bladder cancer.

In head-and-neck cancer, HPV-positive tumors exhibited an almost complete mutual exclusivity with mutations in known drivers such as *TP53*, *CDKN2A* and *TERT* (FDR-corrected *P* *=* 1.73 × 10^−5^, 1.73 × 10^−5^ and 0.012, respectively; multiple testing corrected for presented mutations in EBV and HPV, DISCOVER^22^) (Fig. 3c, Supplementary Table 13), as reported previously^25^, which could be explained by the mutation-independent inactivation of TP53 due to the human papillomaviruses^28,29,30^. Furthermore, we found that mutational signature 2 was enriched in alphapapillomavirus-positive cases of head-and-neck cancer^31^ (FDR-corrected *P* *=* 0.02; Fig. 3d, Supplementary Tables 12, 22). In addition, the expression of APOBEC3B is significantly higher in virus-positive head-and-neck cancers compared with virus-negative cancers^32^ (*P* = 1.6 × 10^−4^; Fig. 3f). However, we did not observe enrichment of APOBEC signatures and changes in expression in EBV-positive samples found in the cervix or in other tissues.

Distinct expression profiles between virus-positive and virus-negative tumors in head-and-neck cancer were observed^33^ (Fig. 3e, Supplementary Table 23). Analyzing the immune cells estimated by CIBERSORT, we identified a significant increase in macrophages and T-cell signals in alphapapillomavirus-positive head-and-neck cancers (*P* *=* 0.004, 0.012 and 0.012 for follicular helper, CD8 and regulatory T cells, respectively, and *P* *=* 0.018 for M1 macrophages; FDR corrected for all viruses and cell types tested; Fig. 3g, Supplementary Table 24). Our integrative analysis of HPV reconfirms many of the findings related to HPV infection, illustrating the potential of our systematic approach in identifying and characterizing tumor-associated viruses.

### Activation of endogenous retroviruses linked to outcome

**Fig. 4:**
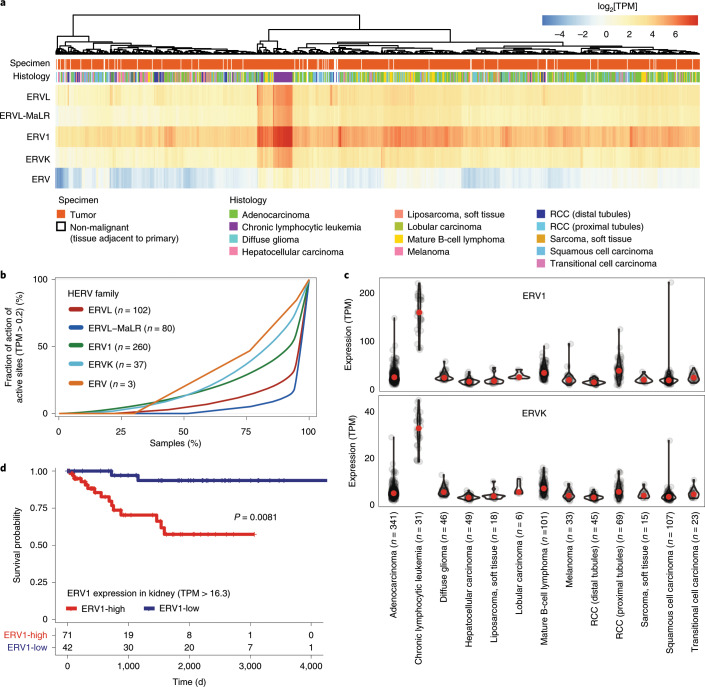
Expression of ERVs. **a**, Heat map showing the expression of HERV across all tumor samples. HERV transcripts per million (TPM) were grouped by family and summed up. Hierarchical clustering was performed by family according to Manhattan distance with complete linkage after log_2_ transformation of HERV TPM expression values.(RCC, renal cell carcinoma). **b**, Fraction of active loci in the genome with a TPM > 0.2 plotted against the fraction of samples. **c**, TPM-based expression of the highly expressed HERVs ERV1 and ERVK across tumor types. *n*, number of analyzed tumor samples. Violin plots are shown; red dots indicate the median. The upper and lower boundaries of the violin plot extend to the maximum and minimum values. **d**, Survival difference between patients with kidney cancer expressing high (red) and low levels (blue) of ERV1. Kaplan–Meier curve shows the overall survival of patients (*n* = 113) with high and low levels of ERV1 with a cut-off of 16.3 TPM (log-rank test *P* = 0.0081). The number of patients at risk is shown at the bottom.

**Extended Data Fig. 5:**
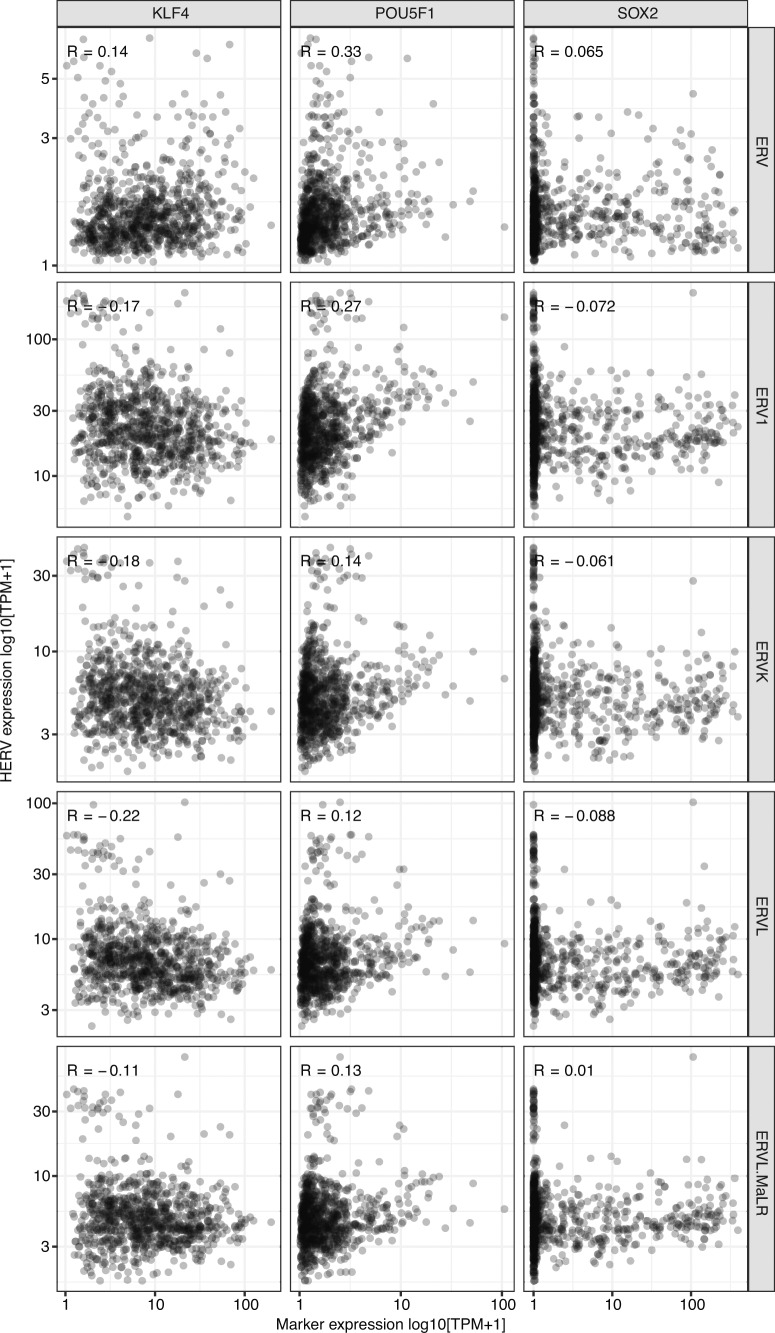
Expression of stem cell markers in relation to HERV expression. Expression values of KLF4, POU5F1 and SOX2 in relation to transcriptional activity of HERVs (ERV, ERV1, ERVK, ERVL, ERVL.MaLR) for 908 tumor samples. Correlation coefficient (R) presented is calculated using Spearman Rank Correlation.

**Extended Data Fig. 6:**
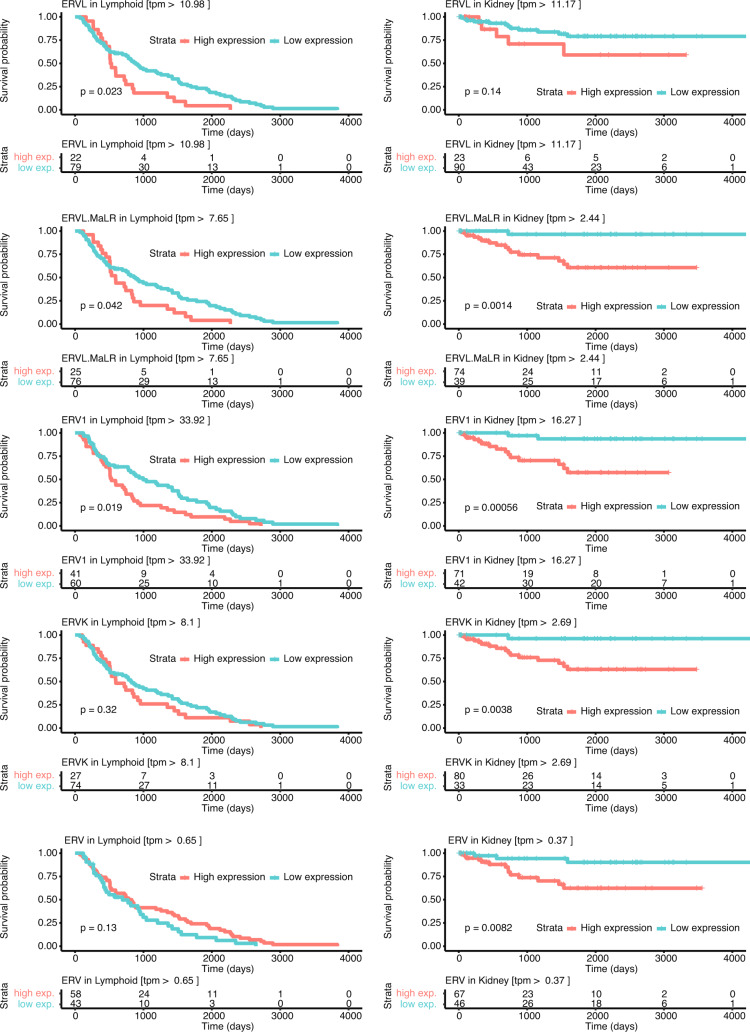
Overall Survival analysis of endogenous retrovirus expression in different tissue types. Cut-offs were defined by maxstat R package using log-rank test and. P values were corrected for multiple testing of variable cut-offs using Lau2 method. Analyzed were all tissue types with more than 40 cases and at least 15 events. Number of patients at risk is provided separated by high or low expression groupings based on the tpm cutoff for the respective ERV family provided in the title of individual panels. P-value of the log-rank test is provided for each analysis.

Human endogenous retroviruses (HERV) are integrations in the human DNA that originate from infection of germline cells by retroviruses over millions of years^34^ and contribute over 500,000 individual sites, or 2.7% of the overall sequence the human genome^35,36^. ERVs were identified by all three pathogen-detection pipelines but were filtered by CaPSID and SEPATH. In addition, an alignment-based approach was used to detect HERV sequences that were embedded in the human reference genome that could be missed by the pipelines by focusing only on non-human reads. In this study, we quantified the expression of HERV-like long terminal repeat retrotransposons that were categorized into several clades by Repbase^37^ as ERVL, ERVL-MaLR, ERV1, ERVK and ERV (Supplementary Table 14). In comparison to the other HERV families, ERV1 shows the strongest expression on average (Fig. 4a) and ERVK the highest fraction of active loci (Fig. 4b). By analyzing the expression of HERVs, we could identify strong expression of ERV1 in chronic lymphocytic leukemia compared with all other tumor tissues and adjacent normal tissues (Fig. 4c). However, we could not identify a link between transcriptionally active stemness markers (OCT3/4, SOX2 and KLF4) and increased HERV expression, in contrast to a previous report^38^ (Spearman rank correlation <0.35; Extended Data Fig. 5). New data suggest that expression of HERVs is associated with prognosis in clear cell renal cell carcinoma^39^. Analyzing HERV expression in relation to patient survival, we found that high ERV1 expression in kidney cancer was linked to worse survival outcome (*P* *=* 0.0081; log-rank test; Fig. 4d, Extended Data Fig. 6, Supplementary Table 15).

### Genomic integration of viral sequences

Viral integration into the host genome has been shown to be a causal mechanism that can lead to the development of cancer^40^. This process is well-established for HPVs in cervical, head-and-neck and several other carcinomas, and for HBV in liver cancer^41,42^.

**Extended Data Fig. 7:**
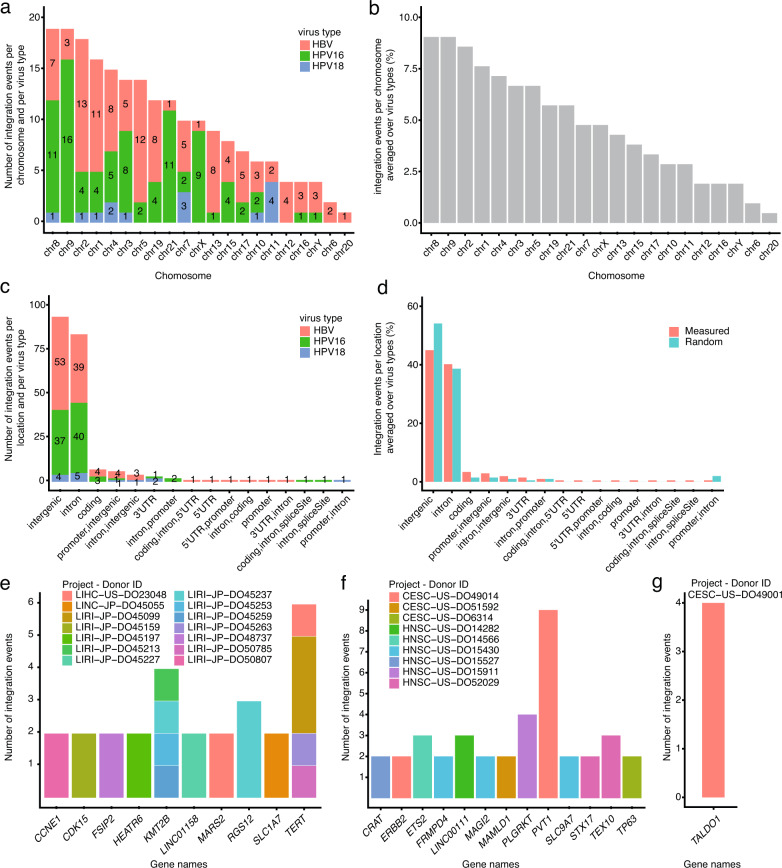
Number of viral integration events as a function of the chromosome and genomic location. **a**, Shows the number of viral integration events detected for HBV, HPV16 and HPV18 as a function of the human chromosome. Numbers within each stacked bar plot represent the number of integration events detected for each virus and within each chromosome. **b**, Shows the percentage of the total number of integration events detected for each chromosome averaged over three viral types shown in panel A. **c**, Number of viral integration events detected for HBV, HPV16 and HPV18 as a function of the host’s genomic location. Numbers within each stacked bar plot represent the number of integration events detected for each virus and within each genomic location. **d**, Shows the percentage of the total number of integration events detected within each genomic location averaged over three viral types shown in panel C. **e**, Shows the number of HBV integration events detected in liver cancers in the host’s gene coding and/or gene promoter regions. Stacked bar plot represents the number of integration events detected within each sample and each gene, each sample is indicated using the color code shown in the legend to the right. **f**, Shows the number of HPV18 integration events detected in head/neck and cervical cancers in the host’s gene coding or gene promoter regions. **g**, Shows the number of HPV16 integration events detected in head/neck and cervical cancers in the host’s gene coding and/or gene promoter regions.

Low-confidence integration events were detected for HHV4 (gastric cancer and malignant lymphoma) and HPV6b (head-and-neck and bladder carcinoma), whereas integration events with high confidence were demonstrated for HBV (liver cancer), AAV2 (liver), HPV16 and HPV18 (in both cervical and head-and-neck carcinoma). Most of these integration events were found to be distributed across chromosomes and a significant number of viral integrations occurred in the intronic (40%) regions whereas only 3.4% of integrations was detected in gene coding regions (*n* = 84 intronic versus *n* = 31 other regions excluding intergenic regions, two-sample test for equality of proportions, *P* = 7.0 × 10^–12^; Extended Data Fig. 7a–d).

**Fig. 5:**
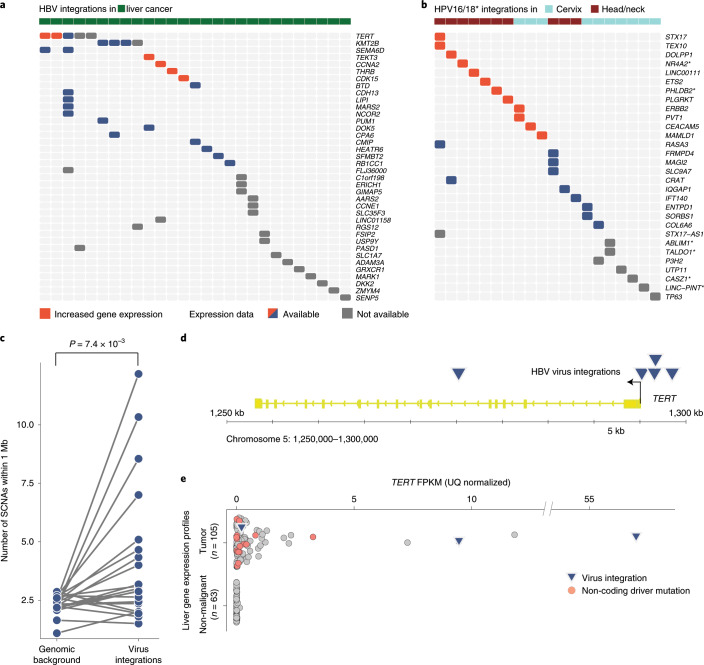
The effect of virus integration. **a**, Integration sites detected in gene regions (including promoter, exon, intron and 5′ UTR regions) are labeled in red for increased gene expression and blue for expression measured. Rows of each heat map designate the nearest genes to the integration sites, and columns represent individual ICGC donor and project IDs. Intragenic HBV integration sites detected in liver cancers (ICGC project codes: LIRI, LIHC and LINC). For *TERT* and *SEMA6D*, intergenic integrations are also shown. **b**, Integration sites detected for HPV16 and HPV18 in head-and-neck (magenta) and cervical (blue) cancers (ICGC project codes: HNSC and CESC). Gene labels with an asterisk indicate HPV18 as opposed to HPV16 viral integrations. **c**, A local increase in the number of SCNAs was shown in the vicinity of HBV integrations (*n* = 21 viral integrations in individual patients, *P* *=* 7.4 × 10^−3^; two-sided paired *t*-test). **d**, Genomic visualization of the HBV integration sites relative to the *TERT* gene in five patients with liver tumors. **e**, The increased gene expression (in FPKM, upper-quartile normalization, UQ) of *TERT* in two liver tumors with HBV integrations in comparison to the expression of *TERT* in tumor and non-malignant adjacent tissues. Tumor samples with a non-coding driver mutation are labeled in orange.

HBV was found to be integrated in 36 liver cancer specimens out of 61 patients who were identified to be HBV positive. Notably, genomic clusters of viral integrations were identified in *TERT* (number of integration sites within a genomic cluster (NGC) of 6), *KMT2B* (NGC = 4)—which was recently identified to be a likely cancer driver gene^43,44^—and *RGS12* (NGC = 3) (Extended Data Fig. 7e). Furthermore, two or more integration events in individual samples were observed in the gene (or gene promoter) regions of *CCNE1*, *CDK15*, *FSIP2*, *HEATR6*, *LINC01158* (also known as *PANTR1*), *MARS2* and *SLC1A7* (Fig. 5a). Additional events with two integration sites were also detected within a distance of 50 kb from *CLMP*, *CNTNAP2* and *LINC00359* genes. Integration events at *TERT* were found to recur in five different liver cancer samples. One sample had a genomic cluster of three viral integration events within *TERT* and four samples contained a single integration event in the *TERT* promoter, or 3′ or 5′ untranslated regions (UTR) (Supplementary Table 17). When comparing gene expression in samples with virus integration to those without, we found that only TERT was overexpressed (fold change ≥2.0) in two liver cancer samples (Fig. 5e). Additional genes with increased expression that were influenced by integration events include *TEKT3*, *CCNA2*, *CDK15* and *THRB* (Fig. 5a).

**Extended Data Fig. 8:**
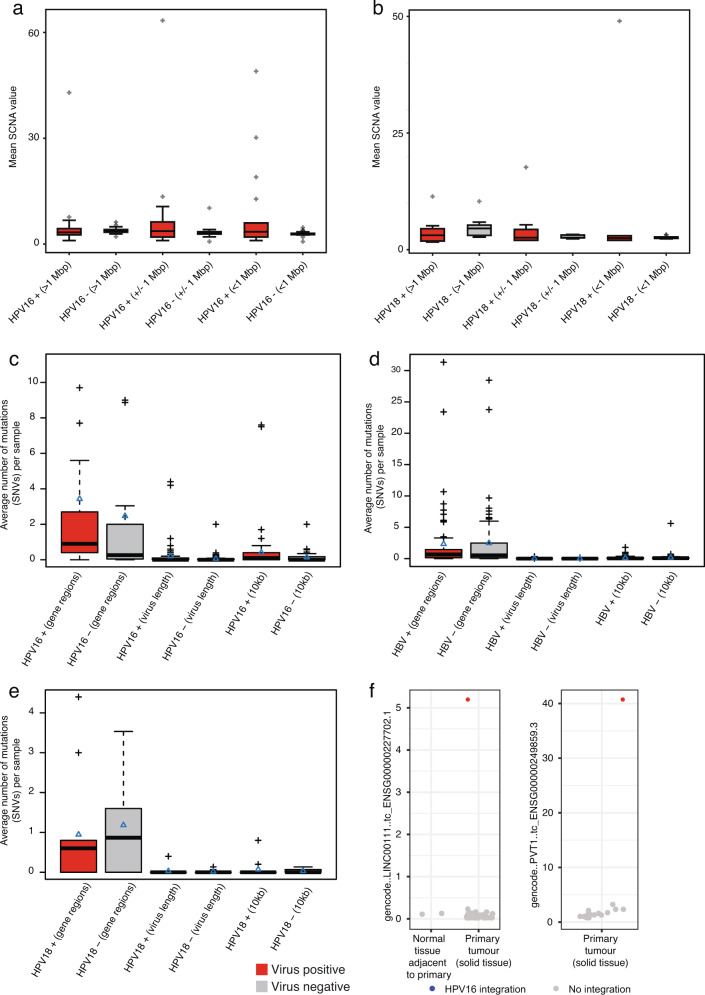
Comparison of the somatic copy number alterations (SCNA) and single nucleotide variants (SNVs) for samples with and without HPV and HBV integrations into human genome. **a**, Boxplots showing the number of SCNA detected in head/neck and cervical cancers: HPV16+(red) vs HPV16- (grey) samples. SCNAs are calculated using three different distances from the integration site: i) greater than 1 Megabases (Mbp), ii) exactly +/− 1 Mbp away, and iii) below 1 Mbp (n = 17 virus integrations). **b**, Boxplots showing the number of SCNAs detected in head/neck and cervical cancers with and without HPV18 integrations (n = 8 virus integrations). **c**, Number of SNVs detected in head/neck and cervical cancers with and without HPV16 integrations. Number of SNVs are calculated using three different ranges for the human genome: i) SNVs within the nearest gene to the virus integration site (maximum: 50Kb), ii) SNVs at the location of the viral integration site in the chromosomal region +/− the position of the second breakpoint located in the viral sequence, and iii) SNVs around 10 kb of the viral integration site. Blue triangles indicate the mean values. (n = 87 virus integrations) **d**, Number of SNVs detected in liver cancers with and without HBV integrations (n = 109 virus integrations). **e**. Number of SNVs detected in head/neck and cervical cancers with and without HPV18 integrations (n = 14 virus integrations). In all Tukey boxplots, black line in the middle represents median and the 25–75th percentiles by the box. The whiskers were drawn up to the 1.5 interquartile range from the lower and upper quartile. **f**, Expression of tumors and normal samples for long noncoding RNAs with and without HPV16 integrations near to the integration site.

There was a significant association between HBV viral integrations and somatic copy-number alterations (SCNAs, Fig. 5c). For samples with HBV integration events, the number of SCNAs was higher on average in the vicinity of viral integration sites (within 1 Mb) compared with samples without HBV integration (mean 4.2 versus 2.3, *P* *=* 7.4 × 10^−3^; two-sided paired *t*-test). No evidence of an SCNA association was seen for other integrated viruses like HPV16 and HPV18 (Extended Data Fig. 8a,b).

HPV18 integration events were detected in seven tumors in total (Fig. 5b), with the most notable clusters of integration events that affected *TALDO1* (NGC = 4) in cervical cancer samples (Extended Data Fig. 7g).

In 20 samples, HPV16 integration events were detected. Genomic clusters of viral integration sites were identified in cervical and head-and-neck cancer samples (Extended Data Fig. 7f). None of these multiple integration events were observed to recur across patients (Fig. 5b). Integration events were also observed in two different long noncoding RNAs (lncRNAs), *LINC00111* and the plasmacytoma variant translocation 1 gene (*PVT1*), an oncogenic lncRNA^45,46^. Expression of both genes is strongly increased in the cases with HPV16 integration (Extended Data Fig. 8f, Supplementary Table 17).

Using the PCAWG SNV calls^10^, we found a significant increase in the number of mutations that occurred within ±10,000 bp of high-confidence viral integration sites (average number of mutations per sample, 0.41 (HPV16^+^) versus 0.14 (HPV16^−^), *P* *=* 0.02; one-sided paired *t*-test, alternative greater, Extended Data Fig. 8c,d). Notably, the integration sites are—compared with a random genome background—enriched in proximity (<1,000 bp) to common fragile sites (*P* *=* 0.0018, Kolmogorov–Smirnov test). These results suggest that HPV16 integration reflects either characteristics of chromatin features that favor viral integration, such as fragile sites or regions with limited access to DNA repair complexes, or the influence of integrated HPV16 on the host genome. Such a correlation was not seen for the integration sites of other viruses (Extended Data Fig. 8e). Finally, a single AAV2 integration event located in the intronic region of the cancer driver gene *KMT2B* ^47^ was detected in one liver cancer sample.

### Identification of novel viral species or strains

**Extended Data Fig. 9:**
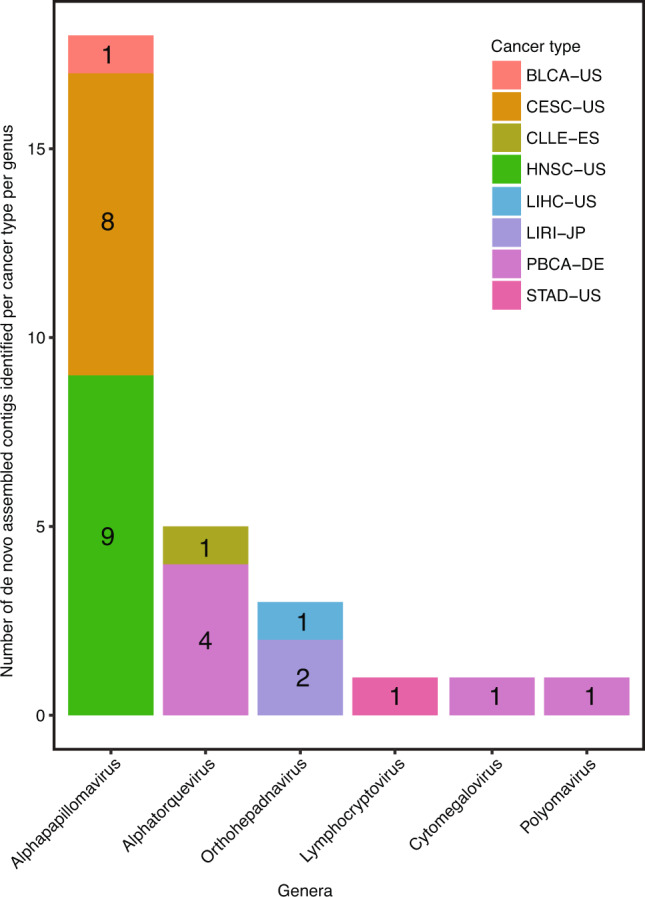
Contigs from de novo assembly identified as possibly originating from novel viral species or strains. Barplot showing the number of contigs obtained using the CaPSID’s de novo assembly step (see Methods) within each genus. Taxonomic classification for each contig was performed using the CSSSCL algorithm. Each of the 29 contigs considered for this plot had to have a sequence homology <90% when aligned to any known sequence contained by the latest nucleotide BLAST database. The legend to the right indicates the following ICGC project codes: BLCA—bladder cancer, CESC—cervical cancer, CLLE—chronic lymphocytic leukemia, HNSC—head and neck, LIHC and LIRI—liver cancer, PBCA—pediatric brain cancer, and STAD—stomach cancer.

De novo analysis using the CaPSID pipeline has generated 56 different contigs that have been classified into taxonomic groups at the genus level by CSSSCL^48^. After filtering de novo contigs for their homology to known reference sequences, we identified 29 contigs in 28 different tumor samples that showed low sequence similarity (on average 63%) to any nucleotide sequence contained in the BLAST database. In this respect, our analysis has shown that WGS and RNA-seq can be used to identify isolates from potentially new viral species. However, the total numbers of novel isolates were low in comparison to viral hits to well-defined genera (Fig. 2c). These de novo contigs were not enriched for a specific tumor entity but rather were distributed across cancer types including bladder, head-and-neck and cervical cancers (Extended Data Fig. 9).

## Discussion

Searching large pan-cancer genome and whole-transcriptome datasets enabled the identification of a high percentage of virus-associated cases (16%). In particular, analysis of tumor genomes, which were sequenced on average to a depth of at least 30-fold coverage, identified considerably more virus-positive cases than investigations of whole-transcriptome data alone, which is the search space analyzed in most previous virome studies. This is probably mainly due to viruses with no or only weak transcriptional activity in the given tumor tissue. Co-infections, generally believed to indicate a weak immune system, were very rare (Extended Data Fig. 3d). This could, however, also be the result of selection processes during tumorigenesis.

Although universal criteria for a causality of viral pathogens are prone to errors, it is worthwhile to look at individual features that might support a potentially pathomechanistic contribution of a given pathogen. These include aspects that affect the expression of host factors (for example, after viral integration) or the mutual exclusivity of the presence of viral genomes and other host factors, which are already known to have a role in the etiology of a given tumor type. Such aspects need to be carefully considered when discussing what strengthens the potentially pathogenic role of a virus.

Not surprisingly, known tumor-associated viruses, such as EBV, HBV, HPV16 and HPV18, were among the most frequently detected targets. Notably, viral detection based on WGS showed similar performance with respect to precision and recall as a targeted PCR for HBV, indicating that this approach is sensitive to detect viruses. This is particularly true for the common integration verified for HBV, HPV16 and HPV18 in our study. In addition, the common theme of potential pathomechanistic effects by the genomic integration of viruses, which were also supported by the observations of multiple nearby integration sites in a given tumor genome that we report in the present study, has gained further momentum. By analyzing the effect of viral integrations on gene expression, we identified several links to genes nearby the integration site. In this regard, the frequently observed integration of HBV at the *TERT* promoter accompanied with the transcriptional upregulation of *TERT* constitutes an intriguing mechanistic example, as the increased activity of TERT is a well-understood driver of carcinogenesis^49^. Furthermore, we also linked viral integrations to increased mutations (SNVs and SCNAs) nearby the integration site.

The known causal role of HPV16 and HPV18 in several tumor entities, which triggered one of the largest measures in cancer prevention, has been the motivation for extensive elucidation of the pathogenetic processes involved. Nevertheless, comprehensive analyses of WGS and RNA-seq datasets revealed additional novel findings. While we confirmed the exclusivity of HPV infection and *TP53*, *CDKN2A* and *TERT* mutations in head-and-neck tumors, we could also link virus presence to an increase in mutations attributed to the mutational signature 2 (ref. ^50^). These are explained by the activity of APOBEC, which—among other effects—changes viral genome sequences as a mechanism of cellular defense against viruses^51,52^. This activation could have an important function in introducing further host genome alterations and, thus, constitute an important mechanism that drives tumorigenesis^32,52^. In liver cancer, mutations in *CTNNB1*, *TP53* and *ARID1A*, major primary oncogenes in this cancer type and HBV infections were confirmed to occur significantly mutually exclusive^23^. Furthermore, the virus-positive head-and-neck cancer samples had a significantly higher abundance of T-cell and M1 macrophage expression signals, which is in agreement with recently described subtypes of head and neck squamous cell carcinoma that differ—among other features—in virus infection and inflammation features.

## Methods

### Identifying potential pathogenic reads

To reduce the number of reads to be considered for the pathogen search, we identified potential pathogenic reads by using P-DiP (https://github.com/mzapatka/p-dip). On the basis of reads aligned to hg19 by BWA^53^ or STAR^54^ using the standard PCAWG approach, we identified read pairs for which at least one read did not map well to the human genome (longest stretch of mapped bases from 20 to 30 bases) and read pairs that were unmapped or mapped to NC_007605 (human herpesvirus 4, which is contained in the 1000 Genomes version of the hg19 human reference genome), and extracted these for further processing. To speed up the extraction, we used bamcollate2 from Biobambam2 (v.2.08) ^55^ as an input stream to the Python script.

### Identification of ERVs

The expression of ERVs was analyzed using RNA-seq data and aligned STAR sequences based on the settings developed within PCAWG (hg19 and Gencode 19). In contrast to the standard pipeline, the reference transcripts from Gencode 19 were enriched by adding HERV locations extracted from RepeatMasker (http://www.repeatmasker.org, rmsk from UCSC, version 17/08/03) and Featurecounts (subread-1.5.3)^56^ applied to identify reads mapping to the modified reference transcripts. Resulting reads counts were converted into TPM according to Wagner et al.^57^.

### The SEPATH pipeline

Our starting point is to take reads that are not mapped to the human genome, using the extracted potentially pathogenic reads. Low quality bases (*q* < 30) were trimmed from the read ends and the TruSeq indexed adapter and TruSeq universal adapter were removed using Cutadapt (v.1.8.1)^58^. Reads less than 32 bp were discarded. Additional filtering was performed to remove reads that contained more than 5% of Ns or those with low complexity (dust method with maximum score of 10) by using Prinseq (v.0.20.3)^59^. Metagenomic Phylogenic Analysis (MetaPhlAn)^60,61^ was then applied to identify and quantify the presence of bacterial and viral populations. MetaPhlAn comes with a curated marker database of around 1 million unique clade-specific marker genes identified from reference genomes (version 2.0 of the database was used). Reads were aligned against the unique marker gene database by using Bowtie2 (v.2.2.1)^62^ with presets set to sensitive. Reads were then counted and normalized giving an estimation of the relative abundance for each level of the phylogenetic tree.

### Detection and analysis of microbial infectious agents by NGS P-DiP

The assembly-based pipeline (P-DiP) was further developed based on a version implemented by M.A. and A.G.^63^. In summary, the pipeline runs preprocessing, assembly and BLAST searches and stores processing details and final results in a postgreSQL database. For the WGS and RNA-seq analyses, we started with the potentially pathogenic reads extracted from the BWA-aligned WGS BAM files. As a first step, reads were trimmed based on quality using trimmomatic. Thereafter, host reads were subtracted by aligning to the human reference genome (WGS: hg19 excluding NC_007605 and hs37d5 and adding phiX; RNA sequencing: Homo_sapiens.GRCh37.dna.primary_assembly) using Bowtie2 (v.2.2.8)^62^. Trinity (v.2.0.6)^64^ was used for the read assembly of WGS reads that were not aligned by Bowtie with sufficient quality (not aligned with --very-fast (-D 5 -R 1 -N 0 -L 22 -i S,0,2.50) to Homo_sapiens.GRCh37.ncrna, Homo_sapiens.GRCh37.cdna.all or PhiX); for the RNA-seq data we applied idba assembler (v.1.1.3)^65^. Assembled contigs were filtered by size (minimal length of 300 bp). Abundance was estimated by remapping all of the reads that did not align to the human reference to the assembled contigs by using Bowtie2. Putative PCR duplicates identified by mapping location were removed from the abundance count. The taxonomic classification of the size-filtered contigs was performed using the BLAST+ package (v.2.2.30)^66^ and nucleotide databases nt (ftp://ftp.ncbi.nlm.nih.gov/blast/db/FASTA/nt.gz, accessed 15 May 2015) and nr (https://ftp.ncbi.nlm.nih.gov/blast/db/FASTA/nr.gz, accessed 20 April 2015). For the extraction of pathogen hits R-scripts were used to filter the BLAST results (https://github.com/mzapatka/p-dip). In summary, for each of the contigs, the best BLAST hits for each segment of the contig were considered and the reads aligning to these segments identified. Potential contaminants were defined based on the taxonomy annotation in NCBI taxonomy. Any taxonomy ID below plasmids (36,549), transposons (2,387), midivariant sequences (31,896), insertion sequences (2,673), artificial sequences (81,077) and synthetic viruses (512,285) was annotated as potential contamination. Segments with higher read counts of these sequences compared to pathogen hits were flagged as contaminants and not further considered.

### CaPSID description of the analysis workflow

The metagenomic analysis pipeline of CaPSID^11^ starts by first processing a BAM file that contains the reads sequenced from a tumor (or normal) sample aligned to the human reference sequence (GRCh37/hg19). Reads that did not map to the human reference were extracted and filtered for low complexity and quality using the SGA^67^ preprocessing module and then aligned in single-end mode using the Bowtie2 aligner^62^ to 5,652 NCBI^68^ viral reference sequences (RefSeq) and a filter sequence reference database composed of 5,242 bacterial and 1,138 fungal reference sequences that were also downloaded from the NCBI. To improve the sensitivity and specificity with which viral sequences were detected, reads that did not map to any reference with Bowtie2 were realigned to the same viral RefSeq database, using the more-sensitive aligner SHRiMP2 in local alignment mode^69^. At the completion of this two-step alignment process, reads that aligned to viral reference sequences were annotated using the information stored in the genome database of CaPSID, which contains full NCBI GenBank and taxa information. Using information from each aligned read, CaPSID then calculates the following four metrics: (1) the total number of reads (or hits) that aligned across any given viral genome, (2) the total number of reads that aligned only across gene regions within any given viral genome, (3) the total coverage across each viral genome and (4) the maximum coverage across any of the genes in a given viral genome.

#### Filtering of viral candidates with low significance

For the analysis of the tumor WGS or RNA-seq samples, CaPSID reports candidate sequences from dozens of different viral genomes, some of which are not related to the cancer phenotype. Some of these reported viral hits are also due to a series of experimental and computational artifacts. To reduce the number of potential false-positive hits, the CaPSID pipeline flags viral genomes that could be the result of artifacts present in the sequencing data or those with no obvious relation to cancer phenotype and that could be filtered in subsequent steps. The following criteria were used to flag and filter for potential viral candidates: (1) flag viral candidates with low coverage, (2) flag bacteriophage viral genome sequences, (3) report only viral candidates with a read composition different from the one expected when generated from the host’s reference GRCh37/hg19 sequence, (4) flag viral candidates that are typically not known to infect humans and those with low read abundance and/or low overall alignment read accuracy.

In the first step, CaPSID flagged viral genomes with low read count and/or coverage using three metrics, including total number of uniquely aligned reads <3, total genome coverage <10% and maximum gene coverage <50%. Viral genomes with low read count can arise as a result of (1) low read/transcript abundance in the human sequenced sample, (2) unspecific alignment between sequenced short reads (for example, low complexity reads) and viral reference sequences and (3) for RNA-seq library preparation in which highly expressed transcripts generally dominate over low abundance targets. To limit the reporting of viral genomes with very low coverage, we chose to flag all genomes for which the maximum gene coverage was <50%. As this lower bound on the maximum gene coverage applied to individual genes and not to the complete viral genome, it appears to be unlikely that viruses with such low coverage are biologically important. The second step in our filtering approach was to flag bacteriophage viral genomes that are most likely not related to any cancer phenotype. Bacteriophages are detected as a result of the presence of bacteria (or bacterial contamination) in human sequenced samples. The third step was used to determine whether the genome coverage observed for each viral candidate was different from the one expected to arise from reads that originated exclusively from the human reference DNA GRCh37/hg19 sequence. To build the CaPSID background model, we used the ART NGS read simulator. The entire GRCh37/hg19 sequence reference file is first fed to the ART^70^ simulator (parameters: art_illumina [Illumina platform] -l [read length=100 bp] -f [the fold of read coverage to be simulated=100] with default values for indels and substitution rates), which then generates single-end (or paired-end) reads and base quality values.

Reads simulated by ART were then aligned to the viral reference sequence database using the same alignment approach for reads that originated from tumor samples (see above). CaPSID then calculated the four metrics for the GRCh37/hg19 background model using the alignment information from simulated reads that aligned to viral reference sequences. The fourth step consisted of flagging viral candidates that were typically not known to infect humans using a dictionary of around 130 terms that we compiled from a database of all viruses known to infect humans. In addition to the above filtering criteria, CaPSID also considered the read abundance associated with each viral candidate sequence (abundance is expressed in terms of aligned reads in parts-per million of total number of unmapped reads) and the average read percentage identity with which reads aligned to a given viral candidate reference sequence.

#### De novo assembly and taxonomic classification of contigs

The purpose of this analysis step is to attempt to characterize potential novel viral sequences at the species or subspecies level. Unaligned reads that could not be aligned to any of the filter/host or viral reference sequences were assembled into contigs using the IDBA algorithm^65^. Assembled contigs were then masked for repeat regions by using RepeatMasker and then filtered for their size and read coverage (contig length ≥500 bp and coverage >5×). Resulting contigs were then assigned to taxonomic groups at the genus level by using the CSSSCL algorithm^48^. Contigs lacking sequence homology to reference sequences contained in the CaPSID or BLAST nucleotide databases with percentage identity <90% were then selected as suggestive of the presence of new viral strains/isolates or species.

### Defining consensus hits

Identification of the consensus hits was achieved by optimizing two features of the individual genus hits: PMER 1 as cut-off (Supplementary Note) and percentage identity >90%. The 90% percentage identity threshold was determined based on our benchmarking study^12^ that indicated that an alignment-based approach can still accurately characterize viral sequences with up to 10% mutation rate (compared with sequences stored in a reference database). Lowering the threshold, with which short reads align to any given reference sequence below 90% identity on average, results in a drop of sequence coverage due to a high attrition rate of aligned reads, lowering the detection rate and thus providing more uncertain characterizations of viral candidates. Notably, there was no difference in the PMER distribution of common hits across the three pipelines, indicating that a common detection cut-off is reasonable (Extended Data Fig. 3b).

The consensus set was restricted to genera that were covered in at least two detection pipelines (Extended Data Fig. 1b). Notably, we could not detect any more hits with high PMER using the unique search space of P-DiP, indicating that almost all of the viral hits from individual pipelines were also screened by another pipeline.

### Virus integration detection analysis

A subset of viral candidates identified to be present in tumor samples by the CaPSID analysis pipeline (parameters used: PMER ≥ 1.1 and genome coverage > simulated background model) was selected for the detection of viral integration events using the VERSE^71^ algorithm. This subset of viruses included: herpesviruses (HHV1, HHV2, HHV4, HHV5, HHV6A/B), simian virus 40 (SV40) and 12 (SV12), human immunodeficiency virus (HIV1), human and simian T-cell lymphotropic virus type 1 (HTLV1 and STLV1), BK polyomavirus (BKP), human parvovirus B19, mouse mammary tumor virus, murine type C retrovirus, Mason–Pfizer monkey virus, HBV, HPV (HPV16, HPV18 and HPV6a) and AAV2. Below we describe the steps used for the viral integration detection analysis.

Viral integration events in the host can be detected by using paired-end NGS technologies that facilitate the detection of genomic rearrangements, as well as gene fusions and novel transcripts. VERSE is capable of determining virus integration sites within a single base resolution by requiring the presence of both chimeric and soft clipped reads. In addition, VERSE improves the detection through customizing reference genomes and was shown to substantially enhance the sensitivity of the detection of virus integration sites^71^. VERSE categorizes its predictions into one of two classes: (1) a high confidence hit with a single base resolution—if there was a sufficient number of soft-clipped reads to support an integration locus so that CREST was able to detect it; or (2) a low confidence hit with a 10-bp resolution for which CREST failed to detect an integration event because of the lack of high-quality soft-clipped reads.

To further limit the false-positive rate associated with viral integration sites, we compared results obtained with VERSE to those from a previous study^72^. Out of 64 WGS liver cancer samples with HBV integration events that were reported previously^72^, 50 were part of the PCAWG dataset analyzed in this study. Of those, 45 out of 50 tested positive for HBV when analyzed by CaPSID (filtering criteria used: PMER ≥ 1.0, genome coverage > host background model and read percentage identity ≥89%). In addition, 50 of these WGS samples had 23 matching whole-transcriptome samples and 22 of these were identified to be positive for HBV by CaPSID (filtering criteria used: maximum gene coverage ≥50%, read percentage identity ≥89% and PMER ≥ 1.0). By combining WGS and RNA-seq tumor samples, 47 out of 50 samples tested positive for HBV when analyzed by CaPSID.

Using VERSE, virus integration sites were detected in 28 out of 47 (60%) of these. This result indicates that for a subset of viral integration events, VERSE might be a more stringent approach compared to previously used methods^72^. This can be explained by the fact that VERSE requires both the presence of paired-end chimeric and soft clipped reads whereas the previously described method^72^ relied only on paired-end reads. To explore these results further, we compared integration sites obtained with VERSE and those described previously^72^ with an overlapping window of 10 bp. Our analysis indicates that among 23 integration sites identified by VERSE in RNA-seq data and that overlap with the previously published results^72^, 91% were classified with high confidence hits and only 9% with low (N total overlap = 23, high = 21 (91%) and low = 2 (9%)). However, a similar result was not observed for integration events found using WGS data (N total overlap = 14, high = 6 (43%), low = 8 (57%)), for which the proportion of integration events classified as high and low was similar.

Thus, our analysis indicates that one important factor for improving the agreement between these two datasets is the confidence level assigned by VERSE to each candidate integration site—but only in the case when integration sites are detected using RNA-seq data. To reduce the potential number of false-positive hits, we decided to use all integration sites predicted by VERSE when these were obtained using WGS data and only high-confidence calls when using RNA-seq data.

### Contaminations

On the basis of the presence of vector sequences in the contig assembled by P-DiP and the background model from CaPSID, we could identify which virus hits originated from common laboratory contaminants or were due to sequence similarities to the human genome. In addition, we filtered known contaminants (see below). For P-DiP, we filtered all hits that did not have more target reads than any artificial sequence (excluding artificial viruses) on an individual contig region. Hits caused by vector and other artificial sequences were identified by analyzing the assembled contigs for combined hits to viral pathogens and artificial sequences. Checking viral hits that occurred at least 40 times in such a contig, we could clearly separate contaminants from viral pathogens.

The gammaretrovirus hits (NCBI taxonomy ID 153135; species, murine leukemia virus) were also marked as artifacts, on the basis of the additional BLAST hits of the corresponding contigs to the *Mus musculus* genome by P-DiP as well as the background model of the CaPSID pipeline, which was designed to limit the number of spurious hits. Most of the frequent virus hits prone to contamination by artificial sequences were lambdalikevirus, alphabaculovirus, microvirus, simplexvirus, hepacivirus, CMV, orthopoxvirus and punalikevirus. However, restricting to at least 1 PMER for the potential virus hit contaminants reduced these to one CMV case.

### Filtering contaminants

We filtered all Microviridae (taxonomy ID 10841) because of the phix174 spike-in used during sequencing. Caudovirales (taxonomy ID 28883), tailed bacteriophages, were removed as they typically infect bacterial hosts. Baculoviridae were filtered because these infect insect cells and are commonly used in the laboratory. The virus coverage was analyzed by aligning the potentially pathogenic reads with BWA-mem to the human hg19 reference genome after adding the respective virus reference sequence that was most frequently detected within the genus. Coverage was thereafter calculated base specific using BEDTools coverage. As we identified EBV in all 14 normal blood controls from ovarian cancer that were EBV immortalized, these were removed from the virus hits.

### Integration of external PCAWG datasets

We tested for mutual exclusivity, for example, between virus detections and driver gene mutations by applying DISCOVER^22^. On the basis of the gene expression data, immune-cell proportions were analyzed by CIBERSORT^14^. For survival analysis, Cox proportional hazards analysis was performed using R libraries ‘survival’ and ‘survminer’ for the figures. The optimal cut points were identified by maxstat using a previously described method^73^ (library maxstat).

### Virus load

The viral load in relation to the human genome equivalents was calculated based on the human bases sequenced (read length × number of reads mapped to the human genomes), tumor sample purity (if available or 100% otherwise) assuming a ploidy of two and using a human genome size of 2,897,310,462 bases (the mappable part of the human genome). This number of human genome equivalents was then related to the viral genome equivalents that were calculated based on the number of identified viral reads, read length and virus genome size.

$$\begin{array}{l}{\rm{tumor}}\,{\rm{genome}}\,{\rm{equivalents}} = \\ \quad \displaystyle\frac{{{\rm{read}}\,{\rm{length}} \times {\rm{number}}\,{\rm{of}}\,{\rm{reads}}\,{\rm{mapped}}\,{\rm{to}}\,{\rm{the}}\,{\rm{human}}\,{\rm{genome}}}}{{{\rm{mappable}}\,{\rm{human}}\,{\rm{genome}}\,{\rm{size \times tumor}}\,{\rm{ploidy}}}} \times {\rm{tumor}}\,{\rm{purity}}\end{array}$$

$${\rm{virus}}\,{\rm{genome}}\,{\rm{equivalents}} = \frac{{{\rm{read}}\,{\rm{length}} \times {\rm{number}}\,{\rm{of}}\,{\rm{viral}}\,{\rm{sequences}}}}{{{\rm{virus}}\,{\rm{genome}}\,{\rm{size}}}}$$

$${\rm{virus}}\,{\rm{load}} = \frac{{{\rm{virus}}\,{\rm{genome}}\,{\rm{equivalents}}}}{{{\rm{tumor}}\,{\rm{genome}}\,{\rm{equivalents}}}}$$

### Human research participants

The ethics oversight for the PCAWG protocol was undertaken by the TCGA Program Office and the Ethics and Governance Committee of the ICGC. Each individual ICGC and TCGA project that contributed data to PCAWG had its own local arrangements for ethics oversight and regulatory alignment.

### Statistics

If not specified otherwise, we used two-sided Wilcoxon rank-sum tests for groups with *n* > 3. Further details can be found in the Nature Research Reporting Summary.

### Reporting Summary

Further information on research design is available in the Nature Research Reporting Summary linked to this article.

## Online content

Any methods, additional references, Nature Research reporting summaries, source data, extended data, supplementary information, acknowledgements, peer review information; details of author contributions and competing interests; and statements of data and code availability are available at 10.1038/s41588-019-0558-9.

## Supplementary information

Supplementary Information Supplementary Note

Reporting Summary

Supplementary Tables Supplementary Tables 1–24

## Data Availability

Somatic and germline variant calls, mutational signatures, subclonal reconstructions, transcript abundance, splice calls and other core data generated by the ICGC/TCGA PCAWG Consortium are described in an associated paper^10^ and are available for download at https://dcc.icgc.org/releases/PCAWG. Additional information on accessing the data, including raw read files, can be found at https://docs.icgc.org/pcawg/data/. In accordance with the data-access policies of the ICGC and TCGA projects, most molecular, clinical and specimen data are in an open tier that does not require access approval. To access potentially identifying information, such as germline alleles and underlying sequencing data, researchers will need to apply to the TCGA Data Access Committee (DAC) via dbGaP (https://dbgap.ncbi.nlm.nih.gov/aa/wga.cgi?page=login) for access to the TCGA portion of the dataset, and to the ICGC Data Access Compliance Office (DACO; http://icgc.org/daco) for the ICGC portion. In addition, to access somatic SNVs derived from TCGA donors, researchers will need to obtain dbGaP authorization. Datasets described specifically in this manuscript can be found in the Supplementary Tables.

## Code availability

The core computational pipelines used by the PCAWG Consortium for alignment, quality control and variant calling are available to the public at https://dockstore.org/search?search=pcawg under the GNU General Public License v.3.0, which enables the reuse and distribution of the pipelines. The pathogen-discovery pipeline P-DiP is available on GitHub (https://github.com/mzapatka/p-dip). CaPSID is available from GitHub (pipeline, https://github.com/capsid/capsid-pipeline; webapp, https://github.com/capsid/capsid-webapp). The taxonomic classifier CSSSCL is available from GitHub (https://github.com/oicr-ibc/cssscl).
